# Defect Spinel Aluminum Molybdenum Sulfide: A Dual‐Function Catalyst for Polysulfide Conversion and Aluminum Intercalation in Aluminum–Sulfur Batteries

**DOI:** 10.1002/advs.202417061

**Published:** 2025-03-24

**Authors:** Qiuping Zhou, Yi Liu, Huayan Zhang, Chenlong Feng, Xinyuan Jiang, Guang Yang, Yongjun He, Ming Chen, Guowang Diao, Lubin Ni

**Affiliations:** ^1^ School of Chemistry & Chemical Engineering Yangzhou University Yangzhou 225002 P. R. China

**Keywords:** Aluminum–sulfur batteries, aluminum intercalation, bimetallic catalysts, polysulfide conversion, spinel sulfide

## Abstract

Aluminum–sulfur batteries (ASBs) are regarded as promising energy storage devices due to their cost‐effectiveness and safety. However, ASBs suffer from problems of polysulfide shuttling and short lifetimes, which restrict their practical applications. In this work, defect spinel Aluminum molybdenum sulfide (AlMo_4_S_8_) embedded in carbon nanotubes synthesized via solid‐state reaction is applied to ASBs. The carbon nanotube‐connected spinel AlMo_4_S_8_ material effectively mitigates polysulfide shuttling while also contributing its own capacity in ASBs. Besides, AlMo_4_S_8_ serves as a “bi‐directional catalyst” with bimetallic active sites to increase the ion transport pathway, effectively facilitating the reduction of polysulfides and the oxidation of Al_2_S_3_. The ASBs with AlMo_4_S_8_/CNTs@S cathode exhibit excellent electrochemical performance with high specific capacity (304.3 mAh g^−1^ at 500 mA g^−1^). The soft pack batteries fabricated with AlMo_4_S_8_/CNTs@S cathode (sulfur loading of 3.0 mg cm^−2^) maintain a stable capacity for more than 50 cycles.

## Graphical abstract

Defective Aluminum Molybdenum sulfides embedded in carbon nanotubes can effectively promote electron transfer by physical confinement and chemical adsorption at the cathode, limiting the shuttling effect of aluminum polysulfides. Also, the bimetallic sulfide itself can contribute to high capacity.

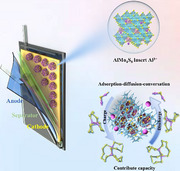

## 1 Introduction

Rechargeable aluminum–sulfur batteries (ASBs) are promising candidates for next‐generation energy storage solutions, offering a high theoretical energy density of 1340 Wh kg^−1^, along with advantages of natural abundance and low cost.^[^ ^1^, ^2^, ^3^ ^]^ Nevertheless, the practical use of ASBs is hindered by voltage lag and poor rate capability.^[^ ^4^, ^5^, ^6^ ^]^ These issues arise from low sulfur utilization and sluggish kinetics, which are attributed to the high energy barrier associated with multi‐electron solid‐state conversion and the dissociation of Al₂Cl₇⁻ into Al^3^⁺.^[^ ^7^, ^8^, ^9^ ^]^ Additionally, the slow conversion reaction between aluminum polysulfide (AlPSs) and Al₂S₃ further exacerbates the problem.

The insulating properties of sulfur are not suitable for direct use as cathode. Recently, much research focused on developing cathode material and separator modifications to accelerate reaction kinetics and mitigate polysulfide shuttling.^[^ ^10^ ^]^ Among them, searching for suitable cathode materials is a key step in advancing the development of high‐performance ASBs. To improve the conductivity and conversion of sulfur, carbon‐based carriers as catalysts have been designed to facilitate the conversion of sulfur.^[^ ^11^, ^12^, ^13^, ^14^ ^]^ However, these nonpolar carbon materials form only weak physical interactions with AlPSs and are ineffective at preventing the dissolution of AlPSs into the electrolyte. Polar compounds (transition metal sulfides,^[^ ^15^, ^16^ ^]^ metal–organic frameworks (MOFs),^[^ ^17^, ^18^ ^]^ and single‐atom electrocatalysts^[^ ^19^, ^20^ ^]^) can adsorb AlPSs by providing “sulfur‐friendly” surface sites through chemical bonds.^[^ ^21^, ^22^ ^]^ However, their limited conversion reactions and low conductivity restrict their further application. Recent studies demonstrated that bimetallic catalysts could enhance the interconversion between AlPSs and Al_2_S_3_, effectively mitigating the shuttling effect. For instance, Zheng et al. reported bimetallic Co/Cu nanoparticles featuring dual active sites that facilitate the efficient adsorption and catalysis of AlPSs.^[^ ^23^ ^]^ Our team subsequently developed Cu‐MoO_2_‐based nanohybrids by pyrolyzing polyoxomolybdate‐based metal–organic frameworks (POMOF) as a cathode material for ASBs.^[^ ^24^ ^]^ The uniform dispersion of Cu within the carbon skeleton, combined with MoO_2_, effectively adsorbs polysulfides. These cathode material designs exhibit remarkably high specific capacitance. However, they contribute to lower gravimetric (*E*_g_) and volumetric energy densities (*E*_v_). If the catalytic host material not only offers additional active sites but also contributes its own capacity, the electrochemical performance and the *E*_g_ and E_v_ of ASBs can be significantly enhanced. Suo and his co‐workers constructed an efficient conducting network through Mo_6_S_8_ to provide high Al^3+^ storage capacity and a strong affinity for polysulfides to inhibit their solvation, achieving excellent electrochemical properties. Meanwhile, transition bimetallic sulfide has attracted research interest. Transition metal sulfides with a spinel structure (sulfur spinel) are important materials in the field of energy storage. In 2018, Xu et al. designed the hollow nanostructure NiCo_2_S_4_, using its secondary structure to construct a microstructured interconnection network that enables both effective trapping of polysulfides and rapid conversion, thereby eliminating the shuttle effect.^[^ ^25^ ^]^ Wang et al. reported CuCo_2_S_4_@CNTs as S host for Li–S batteries. The low bandgap energy and high electrical conductivity of CuCo₂S₄ effectively inhibit the dissolution of polysulfides in the electrolyte.^[^ ^26^ ^]^ AB_4_X_8_ (where A occupy tetrahedral, B occupies the octahedral, and X = S, Se) compounds are known as a family of materials with conductive metal clusters.^[^ ^27^ ^]^ Metal clusters define a collection of a small number of transition metal atoms in which the atoms within the cluster share a common d electron, forming a cluster orbital state like a molecular orbital.^[^ ^28^, ^29^, ^30^ ^]^ Magnetic clusters have at least one unpaired spin, which means that each cluster behaves like a localized moment.^[^ ^31^ ^]^ They have vacancies at each A‐site, leading to tight binding of transition metal cations at the B‐site in tetrahedrally formed B_4_ clusters, and thus can be described as breathing spinels.^[^ ^32^, ^33^ ^]^ Defect engineering has been shown to effectively trap and activate adsorbed reactants, enhancing their catalytic properties. This approach offers a promising pathway for obtaining high‐performance active materials that improve electrochemical performance. Lai et al. reported MoC_x_@NC as S host in Li–S battery, where the defect enhances the active sites to promote electrolyte transport and electron transfer.^[^ ^34^ ^]^ Based on the above characteristics, a new strategy for defect‐based bimetallic sulfur spinel was proposed, which has not been reported in ASBs.

Herein, we synthesized an Aluminum molybdenum sulfide embedded in carbon nanotube with vacancies in a spinel as a sulfur‐hosting material that significantly improves the performance of ASBs. AlMo_4_S_8_ nanosheets exhibit high redox‐catalytic activity, contributing to their own capacity, while the carbon nanotubes offer excellent electrical conductivity, facilitating the conversion reactions of sulfur species in ASBs. Toward verification of this finding, we elucidate the catalyst's mechanism using various spectroscopic methods and in situ electrochemical characterization, complemented by theoretical calculations. Accordingly, the sulfur content of AlMo_4_S_8_/CNTs@S is 63.8 wt.%, contributing to a long cycle life for the ASBs. The unique structure of AlMo_4_S_8_, combined with the enhanced electrical conductivity from the CNTs, accommodates the volumetric expansion of sulfur and promotes efficient ion and electron diffusion.

## 2 Results and Discussion

### 2.1 Synthesis and Characterization

**Figure 1:**
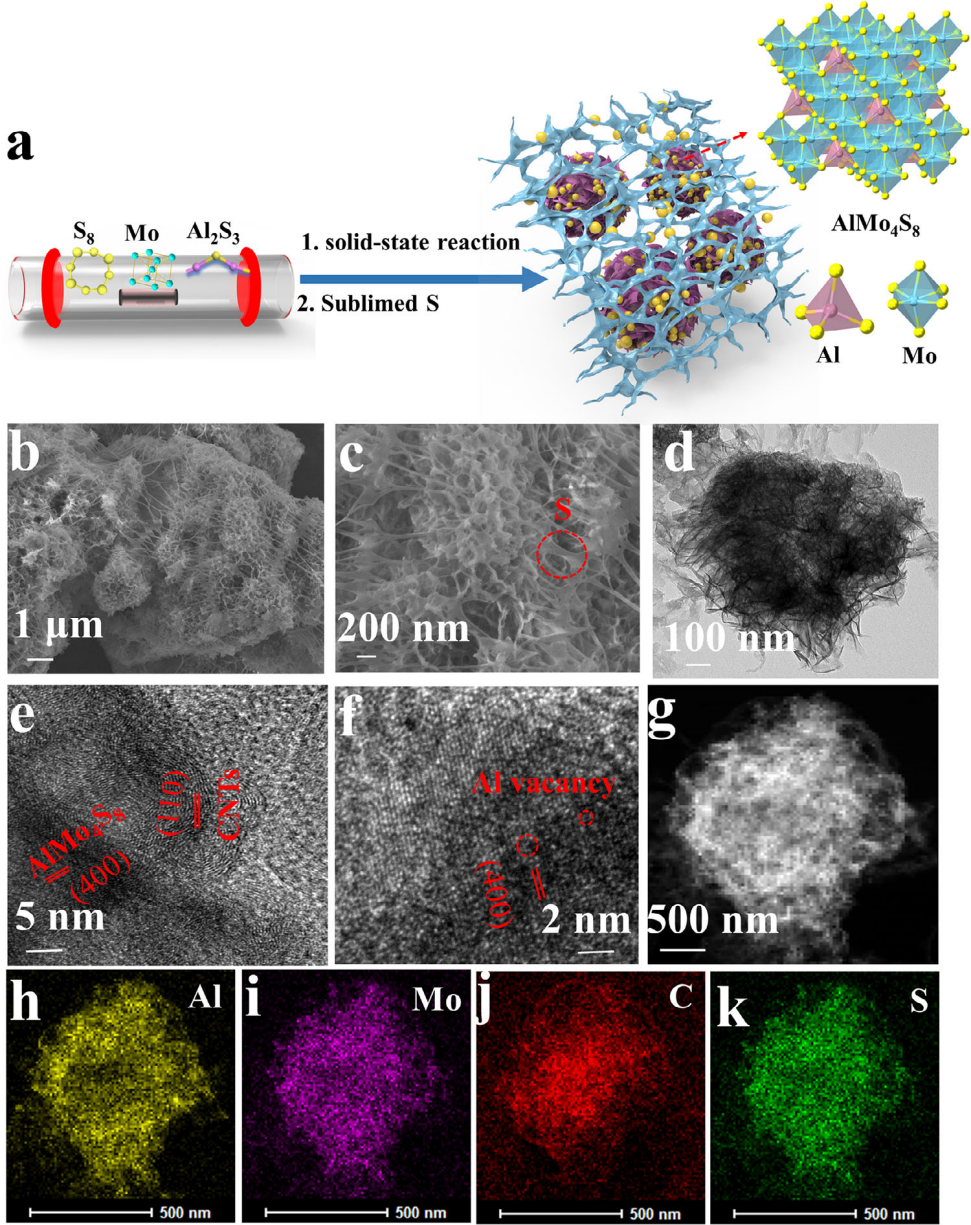
a) Schematic illustration of the fabrication of AlMo_4_S_8_/CNTs@S; b–d) SEM and TEM images of AlMo_4_S_8_/CNTs@S; e,f) High‐resolution TEM (HRTEM) image of AlMo_4_S_8_/CNTs@S; g–k) Dark‐field STEM image and EDX elemental mapping images.

The schematic preparation of AlMo_4_S_8_/CNTs@S is shown in **Figure** **1**a. Molybdenum, sulfur, CNTs, and Al_2_S_3_ were mixed and reacted at high temperature under vacuum. ^[^ ^35^ ^]^ The obtained powder was then stirred in dimethyl sulfoxide. Finally, sulfur was poured into AlMo_4_S_8_/CNTs by solid‐state fusion method to obtain AlMo_4_S_8_/CNTs@S cathode material. Scanning electron microscopy (SEM) and transmission electron microscopy (TEM) images were observed for the AlMo_4_S_8_, AlMo_4_S_8_@S, AlMo_4_S_8_/CNTs, and AlMo_4_S_8_/CNTs@S morphological structure.

AlMo_4_S_8_ exhibits a sheet‐like structure (Figure S1a,b, Supporting Information). Combines with carbon nanotubes to form a filamentary network structure and maintains a nanosheet morphology (Figure S2, Supporting Information). The lamellar structure of AlMo_4_S_8_/CNTs@S is curled and the filamentous structure is obvious after sublimed sulfur (Figure 1b,c). While the AlMo_4_S_8_@S morphology in Figure S3 (Supporting Information) display S block attached to the surface of the lamellar structure without void‐loading S. This indicates that the attached carbon tube effectively prevents the dissolution and shuttle effects of polysulfides by anchoring sulfur, thus reducing the appearance of “dead S”. The TEM image in Figure 1d, AlMo_4_S_8_/CNTs@S exhibits a nanosheet morphology with a diameter of ≈500 nm. The HRTEM for AlMo_4_S_8_/CNTs@S reveals the lattice of CNTs and AlMo_4_S_8_ (Figure 1e,f), the uniform interweaving of CNTs and AlMo_4_S_8_ nanosheets confirms the successful composite. Additionally, the high‐angle annular dark‐field scanning transmission electron microscope (HAADF‐STEM) image and corresponding element mapping images display uniform distribution of S element (Figure 1g–k). Furthermore, the energy‐dispersive X‐ray spectroscopy (EDS) spectra acquired from TEM are presented in Figure S4 (Supporting Information).

**Figure 2:**
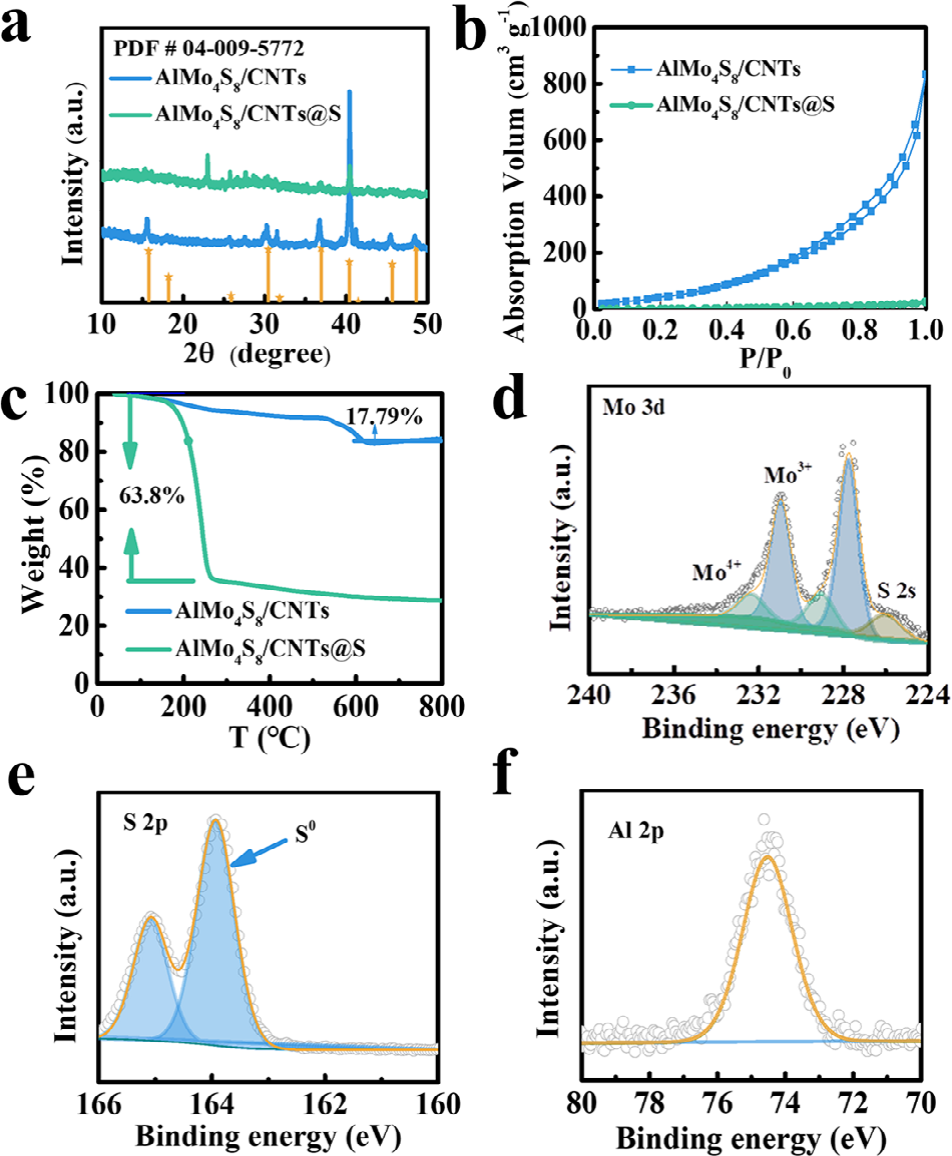
a) XRD patterns of AlMo_4_S_8_/CNTs, and AlMo_4_S_8_/CNTs@S; b) N_2_ adsorption–desorption isotherms for AlMo_4_S_8_/CNTs and AlMo_4_S_8_/CNTs@S; c) TGA curves of AlMo_4_S_8_/CNTs and AlMo_4_S_8_/CNTs@S; d–f) High‐resolution XPS spectra of Mo 3d, S 2p, and Al 2p in the AlMo_4_S_8_/CNTs@S composite.

The XRD pattern (**Figure** **2**a) of the AlMo_4_S_8_/CNTs compound shows the (111), (200), (220), (311), (222), (400), (331), (420), and (422) planes, which match the simulated powder XRD patterns, and that of AlMo_4_S_8_/CNTs@S composite confirms the successful loading of sulfur onto the AlMo_4_S_8_/CNTs surface. Nitrogen adsorption‐desorption isotherms in Figure 2b and Figures S5–S7 (Supporting Information) display the mesoporous nature of AlMo_4_S_8_ and AlMo_4_S_8_/CNTs, with specific surface areas of 150 and 600 m^2^ g^−1^, respectively. Following the sulfur loading, these values decreased to 28 and 30 m^2^ g^−1^, indicating the uniform dispersion of S within the mesopores. The content of S was 63.8 wt.% and CNTs 17.79 wt.% as determined by TGA (Figure 2c). The incorporation of CNTs enhanced the electrical conductivity of the material. The surface composition of the AlMo_4_S_8_/CNTs@S and AlMo_4_S_8_ composites was analysed by x‐ray photoelectron spectroscopy (XPS) (Figure 2d–f; Figure S8–S10, Supporting Information). The Mo 3d_5/2_ and 3d_3/2_ doublets were attributed to Mo^4+^ (232.75, 229.02 eV) and Mo^3+^ (231.09, 227.83 eV) (Figure 2d).^[^ ^36^, ^37^, ^38^ ^]^ The new peaks located at 163.83 and 164.98 eV (Figure 2e) correspond to elemental sulfur (S^0^).^[^ ^22^, ^39^, ^40^ ^]^ The high‐resolution Al 2p XPS spectra, as shown in Figure 2f. The Al 2p peak at 74.5 eV confirms the presence of Al^3+^.^[^ ^41^, ^42^, ^43^ ^]^ XPS spectrum of AlMo_4_S_8_ is consistent with the AlMo_4_S_8_/CNTs@S compound (Figures S8–S10, Supporting Information).

**Figure 3:**
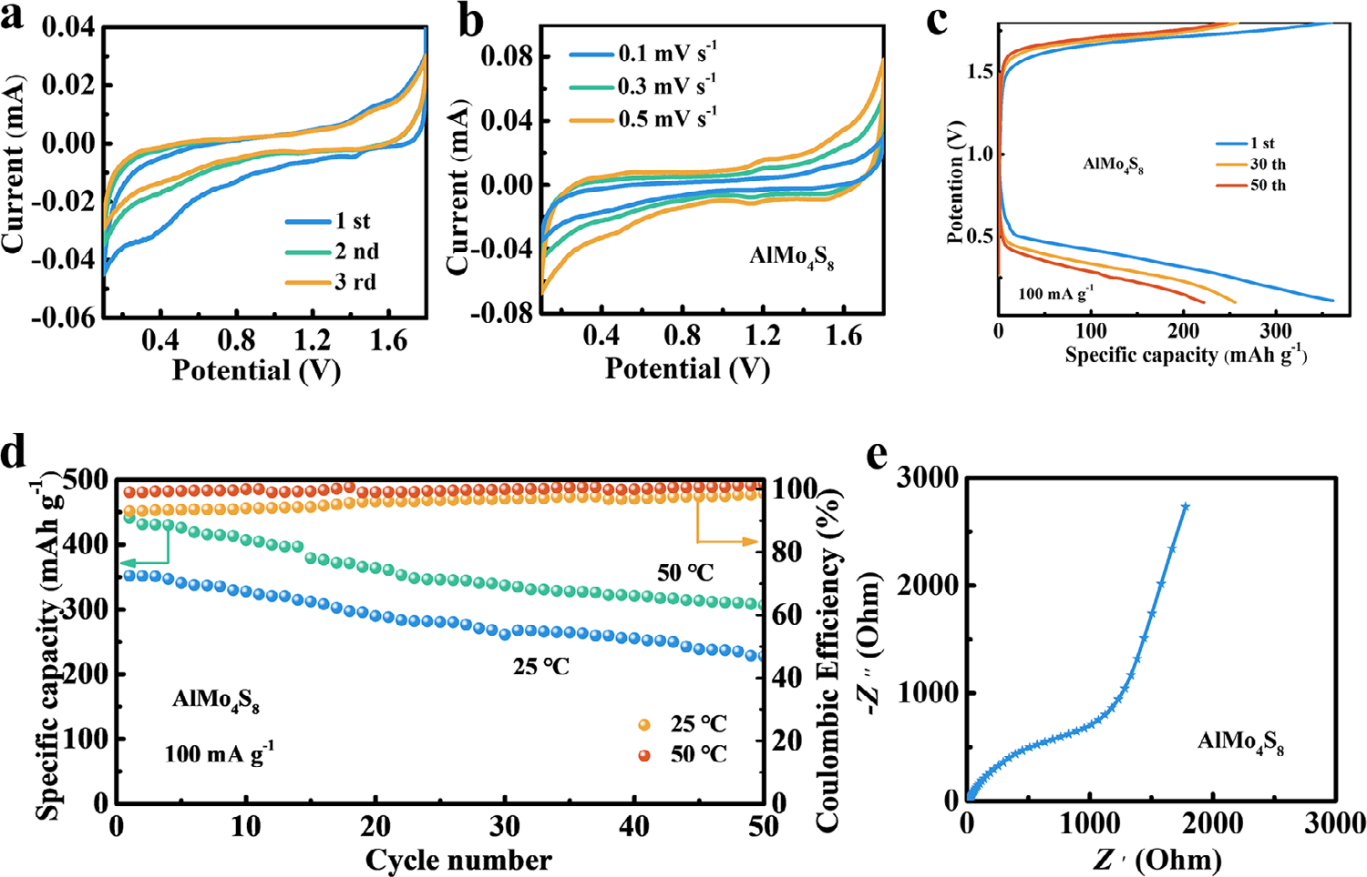
a) CV curves of AlMo_4_S_8_ in the voltage range 0.1–1.8 V for the first three cycles at the scan rate of 0.1 mV s^−1^; b) CV curves of AlMo_4_S_8_ at the different scan rate; c) Charge–Discharge curves of Al‐ion battery with AlMo_4_S_8_ at 100 mA g^−1^ at 25 °C; d) Cycling performance of cathode AlMo_4_S_8_ at 100 mA g^−1^ at 25 and 50 °C; e) Nyquist plots.

The defect spinel AlMo_4_S_8_ can enhance capacity through the incorporation of Al ions. The cyclic voltammetry (CV) profiles shown in **Figure** **3**a for AlMo_4_S_8_//Al half‐cells indicate that multiple redox reactions take place during cycling. The representative cathodic peaks at 1.25, and 0.4 V can be attributed to the insertion of aluminum ions. As the sweep rate increases, the redox peaks become more pronounced, and the cathodic peak exhibits minor polarization, indicating that the AlMo_4_S_8_ structure remains stable and is less likely to degrade during cycling (Figure 3b). We further investigated the relationship between the capacitive contribution and the ion diffusion contribution in electrochemical energy storage. The kinetic behavior observed from CV should adhere to the power law (*i* = a*v* ^b^, where a and b are adjustable parameters, *i* is the current (A), and v is the potential scan rate).^[^ ^44^, ^45^ ^]^ The calculated b value provides a distinction between the capacitive contribution (b = 1) and the ionic diffusion contribution (b = 0.5).^[^ ^46^ ^]^ The power law can be transformed into log(i) = blog(*v*)+blog(a), where the b value is the slope of the fitted line.^[^ ^47^ ^]^ The linear relationship between log(*i*) and log(*v*) is derived from the cathodic current of the CV curve, as shown in Figure S11 (Supporting Information). Notably, the b value of AlMo_4_S_8_ is close to 1, so the Al^3+^ intercalation layer of AlMo_4_S_8_ mainly behaves capacitively during the charging and discharging process. The second cycle charge/discharge curve for Al//AlMo_4_S_8_ displays capacity 380 mAh g^−1^ at 10 mA g^−1^ (Figure S12, Supporting Information). Moreover, the discharge capacity is retained at 260 and 228 mAh g^−1^ for the 30th and 50th cycles at high current density of 100 mA g^−1^ (Figure 3c). The cycling performance of the Al//AlMo_4_S_8_ half‐cell (Figure 3d) presents an initial capacity of up to 350 mAh g^−1^ at 100 mA g^−1^. At a higher temperature of 50 °C, the AlMo_4_S_8_ cathode maintains stable cycling performance, achieving a capacity of 380 mAh g^−1^ after 50 cycles. At high current density of 500 mA g^−1^, the AlMo_4_S_8_ capacity is only 35 mAh g^−1^ (Figure S13, Supporting Information). SEM and TEM analyses reveal that the morphology and lamellar structure of AlMo_4_S_8_ are well‐preserved even after 50 cycles at 100 mA g^−1^ (Figure S14, Supporting Information). Such high cell capacity and cycling stability are attributed to the stable insertion/de‐embedding behavior of Al^3+^ in the AlMo_4_S_8_ metal clusters. Electrochemical impedance spectroscopy (EIS) was further employed to measure the electrical resistance of AlMo_4_S_8_ (Figure 3e; Figure S15, Supporting Information). The charge transfer resistance (*R*_ct_) for AlMo_4_S_8_ electrode was 43 Ω, indicating that the defective AlMo_4_S_8_ spinel significantly enhances charge transfer. Al vacancies promote Al^3+^ intercalation /de‐embedding in Al‐ion battery.

**Figure 4:**
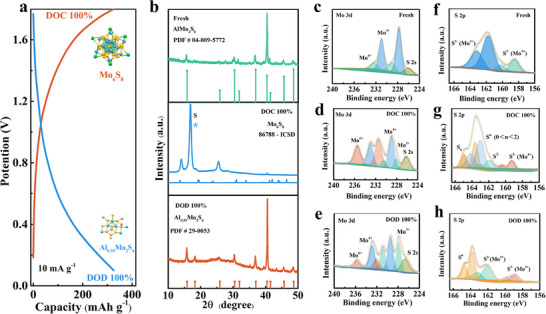
a) Charge–Discharge profiles of Al‐ion battery with AlMo_4_S_8_; b) XRD for the AlMo_4_S_8_ during the electrochemical reaction; X‐ray photoelectron spectroscopy of c–e) Mo 3d, f–h) S 2 of the cathode AlMo_4_S_8_ at various charged/discharged stages.

Further elucidating the AlMo_4_S_8_ intercalation mechanism for aluminum, various states of the AlMo_4_S_8_ electrodes during charge and discharge were selected for XRD and XPS analyses. The battery was prepared using AlMo_4_S_8_ with the conductive agent super p as cathode and metallic aluminum as anode. The cathode material was tested after cycling at 10 mA g^−1^ in three different states (fresh, charged to 1.8 V, and discharged to 0.1 V) (**Figure** **4**a), and the high‐resolution and full spectra of Mo and S elements are shown in Figure 4c–h, respectively. XRD spectra were tested for fully charged and discharged state AlMo_4_S_8_ electrodes (Figure 4b). At fully charged, Al^3+^ was detached from the AlMo_4_S_8_ metal clusters, showing the characteristic peaks of Mo_6_S_8_. At fully discharged state, Al^3+^ was embedded in Mo_6_S_8_ to generate the structure Al_0.55_Mo_2_S_4_. Afterward, the chemical valence changes of AlMo_4_S_8_ in charged and discharged states were measured with the same assembled batteries. In the fully charged AlMo_4_S_8_ electrode, the main doublet strong peaks of Mo^3+^ (228 and 231.05 eV)^[^ ^48^ ^]^ became weaker, the Mo^4+^ (229.5 and 233.5 eV)^[^ ^49^, ^50^ ^]^ peaks enhanced, and the new peaks emerged in high binding energy (the emerging peaks belonging to Mo^6+^ (236.1 eV).^[^ ^51^, ^52^, ^53^ ^]^ At the fully discharged state of the AlMo_4_S_8_ electrode, the peak corresponding to Mo⁶⁺ decreases as it is reduced to Mo⁴⁺. This result is in accordance with the CV results for the reaction to be reversible. The Al 2p signals before and after charging and discharging are shown in Figure S16 (Supporting Information). After fully charging to 1.8 V, the intensity of the Al 2p XPS peak decreases due to the de‐embedding of the Al^3+^ from within the AlMo_4_S_8_ cathode.^[^ ^54^ ^]^ However, when fully discharged to 0.1 V, the signal of Al 2p was enhanced, indicating the insertion of Al^3+^ into the Mo_6_S_8_ cathode.^[^ ^46^ ^]^ This result is consistent with the XRD results, demonstrating that Al^3+^ can be reversibly de‐embedded/inserted into the AlMo_4_S_8_. The reaction equations in cathode electrochemical process are as follows Reaction 1 and 2.

(1) $$\mathrm{Charge}:3\mathrm{AlM}o_{3}S_{8}-3Al^{3+}-9e^{-}\to 2Mo_{6}S_{8}+8S$$

(2) $$\begin{array}{ccc} & & \mathrm{Discharge}:20{\mathrm{Mo}}_{6}S_{8}+80S+33Al^{3+}+99e^{-}\\ & & \to 60Al_{0.55}Mo_{2}S_{4} \end{array}$$

The morphology of AlMo_4_S_8_ electrodes after cycling was also investigated by TEM combined with EDX elemental mapping (Figure S17, Supporting Information). The electrode AlMo_4_S_8_ morphology nanosheets are still present after cycling and the mapping still exhibit Al, Mo, and S elements, which indicates that AlMo_4_S_8_ has high structural stability.

**Figure 5:**
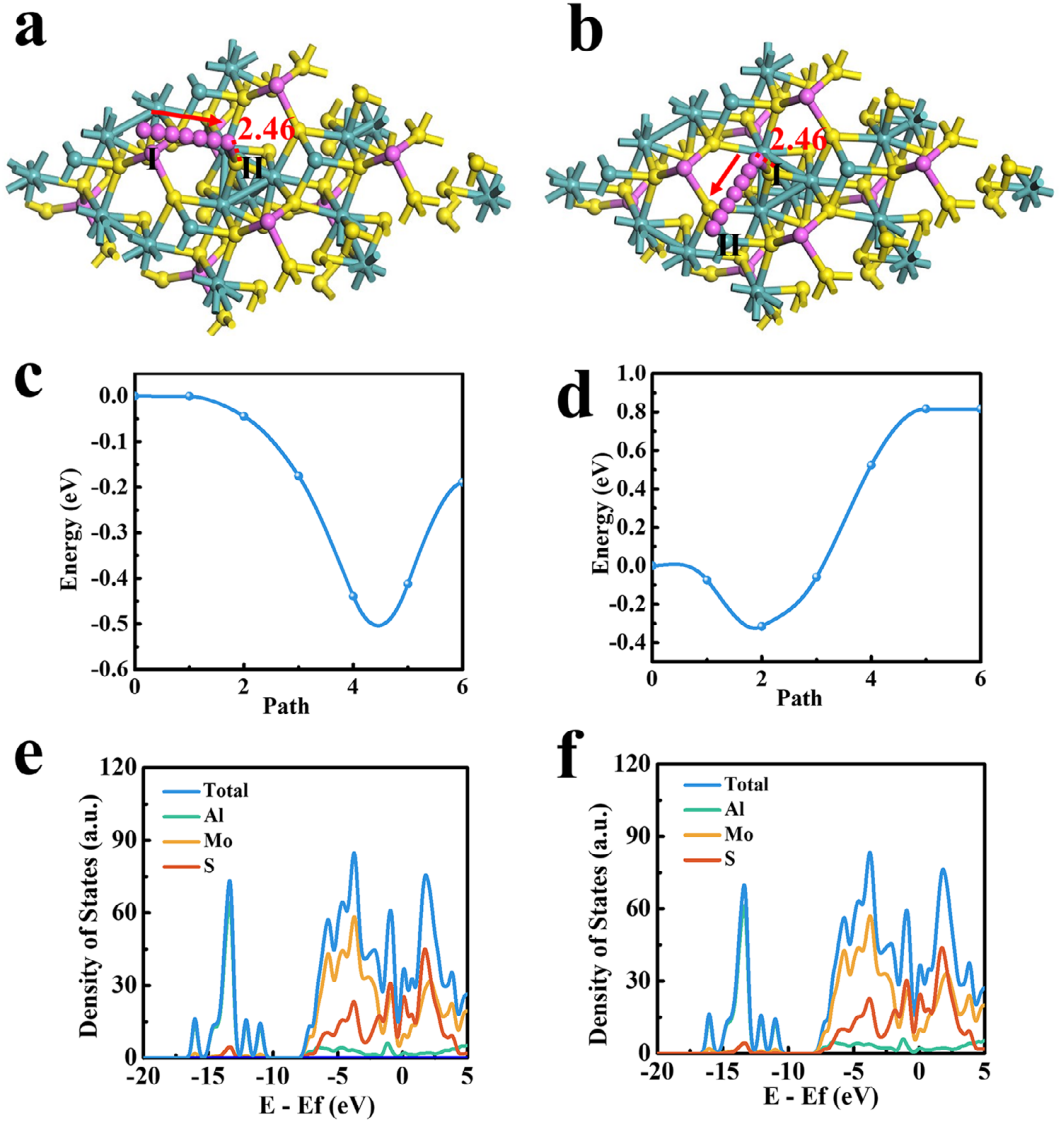
The DFT calculations results, a,b) The interstitial sites in AlMo_4_S_8_; The corresponding migration energies of Al in AlMo_4_S_8_, c) from I (Al) to II (S) and d) from I (Mo) to II (S); e) Total DOS and PDOS for AlMo_4_S_8_; f) Total DOS and PDOS for Al‐ion in AlMo_4_S_8_.

We further conducted density‐functional theory (DFT) calculations to examine the shift paths and corresponding energy barriers of the influence of Al vacancies on the migration of Al. The migration paths and the corresponding migration energies of Al ion in AlMo_4_S_8_ are shown in **Figure** **5**. Two migration paths of Al ions are from I (Al) to II (S) and from I (Mo) to II (S), with migration energies of −0.53 eV (Figure 5c) and −0.21 eV (Figure 5d), respectively. It is evident that the energy barrier of path I (Mo) to II (S) is significantly lower than that of path I (Al) to II (S). Al is active on both the vacancies in AlMo_4_S_8_ and Mo, which result is consistent with the CV. Apart from ion dynamics, the electronic properties of AlMo_4_S_8_ and Al‐ion in AlMo_4_S_8_ were compared by total density of states (TDOS) and partial density of states (PDOS). The unoccupied state accepts electrons upon electrochemical reduction and Al‐ion cation intercalation, with no shift in the PDOS spectrum. The results show that the embedding of Al ions has no effect on the conductive properties of AlMo_4_S_8_ with a stable structure. The above DFT calculations demonstrate the superiority of vacant AlMo_4_S_8_ in terms of ionic/electronic dynamics. The vacancies in AlMo_4_S_8_ will positively improve the transport of Al ion and thus the electrochemical properties.

**Figure 6:**
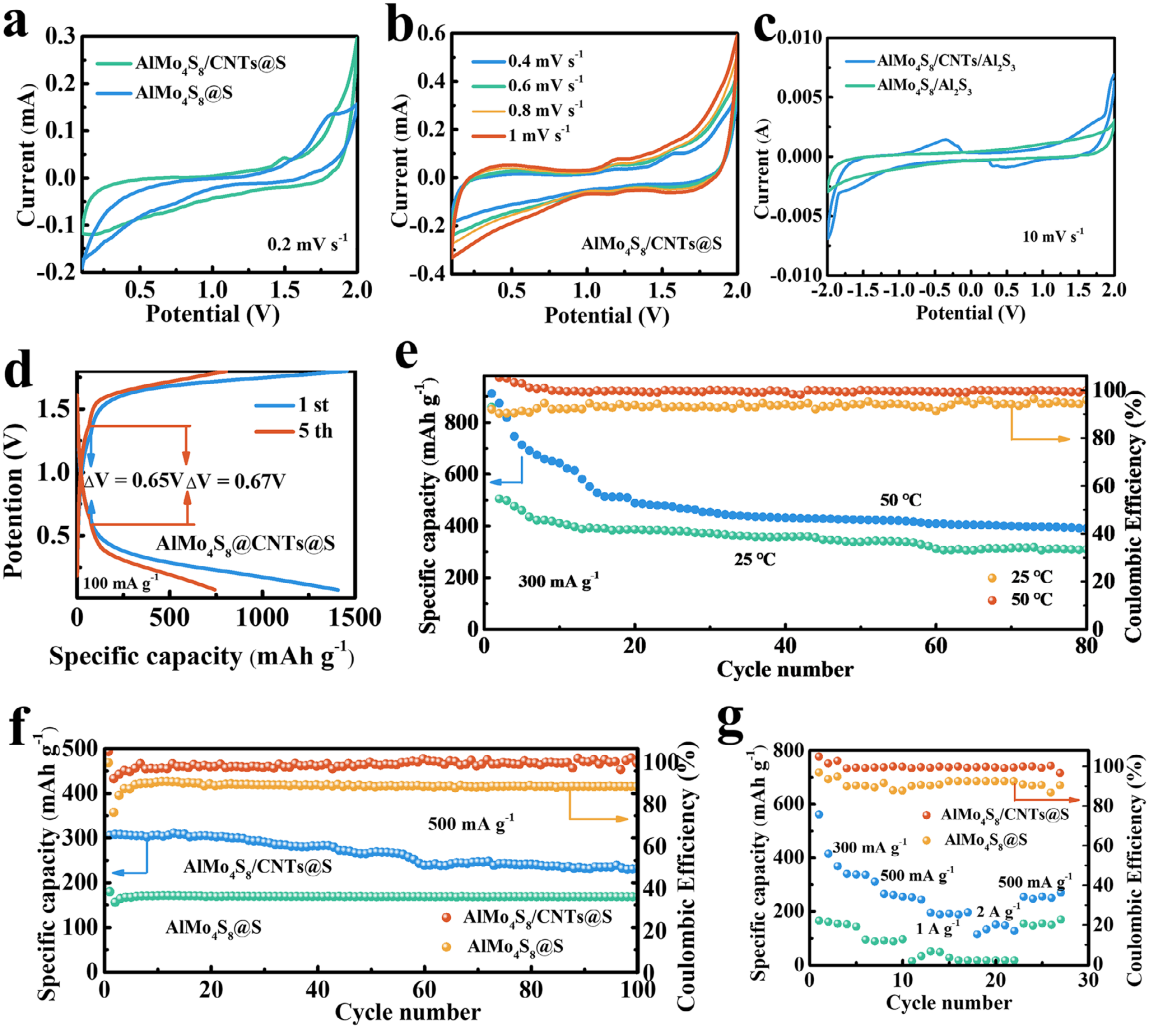
a) CV curves of AlMo_4_S_8_/CNTs@S and AlMo_4_S_8_@S at the scan rate of 0.2 mV s^−1^; b) CV curves of AlMo_4_S_8_/CNTs@S at the different scan rate; c) CV curves of the symmetric cells with Al_2_S_3_; d) Charge–discharge profiles of Al–S battery featuring AlMo_4_S_8_/CNTs@S at 100 mA g^−1^; e) Cycling ability of Al–S battery with AlMo_4_S_8_/CNTs@S at 300 mA g^−1^ at 25 and 50 °C; f) Cycling performance of Al–S battery with AlMo_4_S_8_/CNTs@S at 500 mA g^−1^; g) Rate performance of the AlMo_4_S_8_/CNTs@S electrode.

To evaluate the kinetics of the polysulfide reduction and oxidation reactions on AlMo_4_S_8_/CNTs@S and AlMo_4_S_8_@S, we performed cyclic voltammetry (CV) measurements on electrode AlMo_4_S_8_/CNTs@S and AlMo_4_S_8_@S. According to the cyclic voltammograms (CV) of AlMo_4_S_8_@S and AlMo_4_S_8_/CNTs@S cathode (**Figure** **6**a), the AlMo_4_S_8_/CNTs@S electrode showed a greater current response with higher S utilization. The reduction peaks appeared at 1.75 and 1.22 V, S was reduced. No polarization occurred with increasing sweep rate, indicating good cycling performance of AlMo_4_S_8_/CNTs@S (Figure 6b). Figure S18 (Supporting Information) showed that the capacitive current contribution could not be obtained in absence of CNTs without significant redox peaks. To verify the catalytic effect of AlMo_4_S_8_/CNTs and AlMo_4_S_8_ on Al_2_S_3_, the symmetric cells with AlMo_4_S_8_/CNTs/Al_2_S_3_ as both anode and cathode were investigated. The peak current density of AlMo_4_S_8_/CNTs/Al_2_S_3_ symmetric cells was much higher than that of AlMo_4_S_8_/Al_2_S_3_ cells, indicating that AlMo_4_S_8_/CNTs greatly improved the reaction rate of Al_2_S_3_ (Figure 6c). The cycling ability for battery utilizing AlMo_4_S_8_/CNTs@S composites was evaluated at 100 mA g^−1^. The first cycle capacity was 1400 mAh g^−1^, and the capacity remained at 520 mAh g^−1^ after 80 cycles (Figure S19, Supporting Information). The voltage plateau value difference of the charge/discharge curves of AlMo_4_S_8_/CNTs@S composite at 100 mA g^−1^ was 0.65 V, displaying small polarization (Figure 6d). At high current density of 300 mA g^−1^, the AlMo_4_S_8_/CNTs@S composite showed a high initial discharge capacity of 859.7 mAh g^−1^, and the capacity kept 262 mAh g^−1^ after 60 cycles. (Figure S20, Supporting Information). The capacity decays fast during the initial cycles is mainly due to the dissolution of polysulfides and the activation of S, keeping the capacity stable after full contact with the electrolyte. Notably, this capacity increased to 911.5 mAh g⁻¹ when the temperature was raised to 50 °C (Figure 6e). After 100 cycles, the AlMo_4_S_8_/CNTs@S composites maintained a substantial capacity of 232.6 mAh g⁻¹. The AlMo_4_S_8_/CNTs@S composite exhibited good cycling performance at 500 mA g^−1^ (Figure S21, Supporting Information). In contrast, the AlMo_4_S_8_@S composites only achieved a specific capacity of 168.6 mAh g⁻¹ at the same current density of 500 mA g⁻¹ (Figure 6f). The Coulomb specific efficiencies were all above 99%, indicating that the AlMo_4_S_8_/CNTs composite effectively mitigates the dissolution and shuttle effects of AlPSs in the electrolyte. Next, the multiplicity performance of AlMo_4_S_8_/CNTs@S and AlMo_4_S_8_@S cathode for Al–S batteries in the range of 300 mA g^−1^ to 2 A g^−1^ was also investigated (Figure 6g). The capacity of the AlMo_4_S_8_/CNTs@S cathode was 535 mAh g^−1^ at 300 mA g^−1^. The reversible specific capacity gradually decreased to 303 and 180 mAh g^−1^ as the current was increased to 500 mA g^−1^ and 2 A g^−1^, respectively (Figure S22, Supporting Information). When the current density was switched back to 500 mA g^−1^, the pristine capacity of AlMo_4_S_8_/CNTs@S composites could be largely recovered, indicating the high stability of AlMo_4_S_8_/CNTs@S composite electrode. In contrast, the capacity of the AlMo_4_S_8_@S decayed more with the increase of the current density, and CNTs@S only delivers a capacity of ≈30 mAh g^−1^ (Figure S23, Supporting Information). To further explore the practical application potential of AlMo_4_S_8_/CNTs@S cathode, high sulfur mass loading (3.0 mg cm^−2^) and a soft package with an area of 9 cm^2^ were used. The AlMo_4_S_8_/CNTs@S cathode exhibited good cycling performance with an initial discharge capacity of 136.9 mAh g^−1^ at 50 mA g^−1^ (Figure S24, Supporting Information). It showed highly competitive electrochemical performance compared to other representative metal sulfide/oxide‐based sulfur host materials (Table S1, Supporting Information). The electrochemical impedance spectra (EIS) of AlMo_4_S_8_/CNTs@S and AlMo_4_S_8_@S electrodes at open‐circuit voltage (OCV) are shown in Figure S25 (Supporting Information). The corresponding equivalent circuit is shown in Figure S26 (Supporting Information). The *R*_ct_ value for AlMo_4_S_8_/CNTs@S electrodes is lower than that of AlMo_4_S_8_@S electrodes, demonstrating that the incorporation of CNTs into the AlMo_4_S_8_ enhances electrical conductivity (Table S2, Supporting Information).

**Figure 7:**
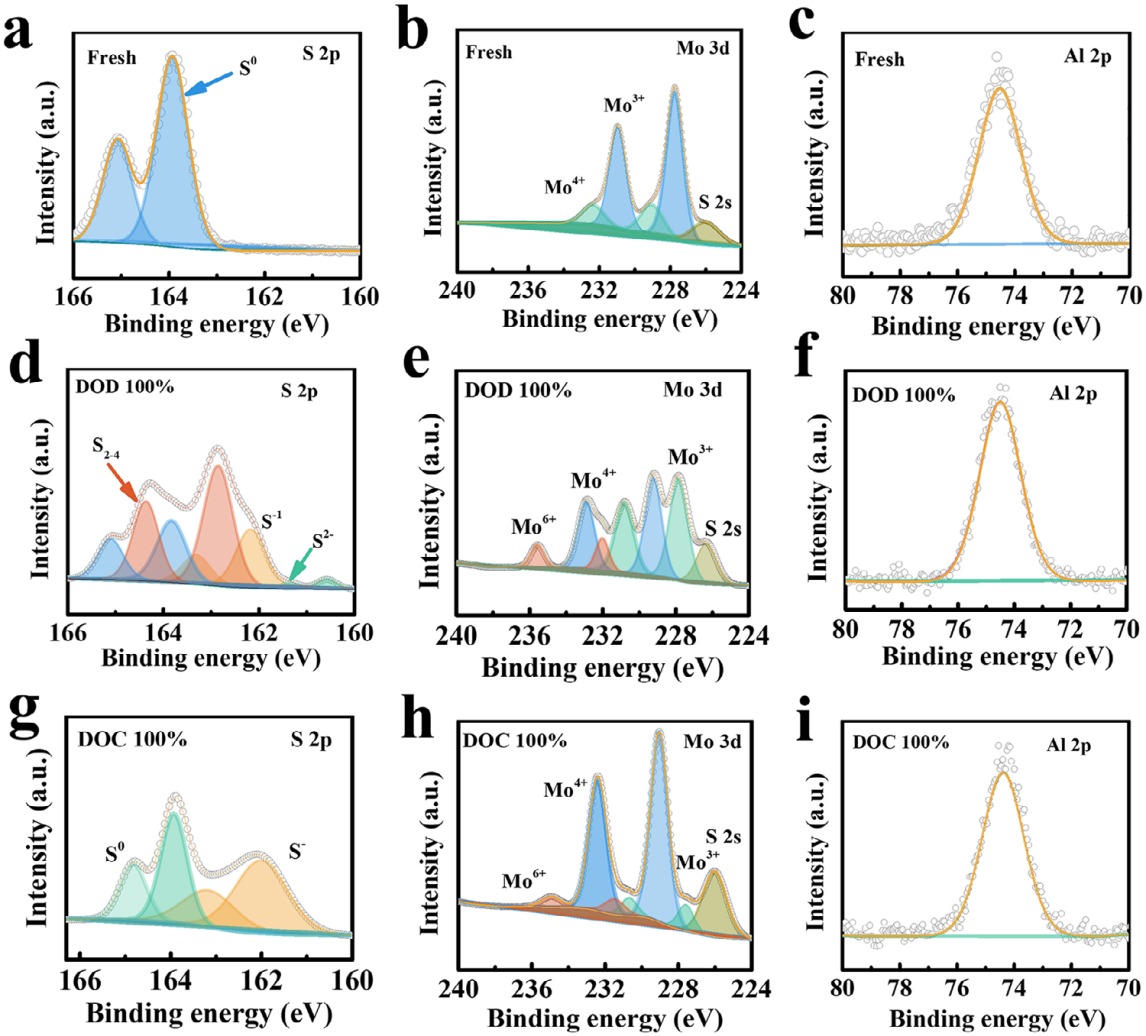
XPS spectra of a,d,g) S 2p, b,e,h) Mo 3d, and c,f,i) Al 2p of the cathode from the AlMo_4_S_8_/CNTs@S//Al batteries at different charged and discharged stages.

To further verify the adsorption capacity of AlMo_4_S_8_/CNTs for AlPSs in Al–S cells, XPS characterization was conducted on AlMo_4_S_8_/CNTs@S electrodes collected from the battery after charging and discharging. **Figure** **7** exhibits the S 2p, Mo 3d, and Al 2p XPS spectra for the AlMo_4_S_8_/CNTs@S cathode in the Al–S cell. The S signals in the AlMo_4_S_8_/CNTs@S composite gradually weakened during the discharge process, while S^2−^ (160.3 eV) and S_2‐4_ (162.85 eV) signals emerged, indicating the formation of short‐chain S and Al_2_S_3_.^[^ ^55^, ^56^, ^57^ ^]^ In contrast, the S electrode exhibited only a partial conversion of S_0_ into S^2−^ during the cycling process, indicating that the material AlMo_4_S_8_/CNTs promotes the interconversion of S and polysulfides, which improves the utilization of S (Figure S27, Supporting Information). Throughout the following charging process, the valence state of the sulfur species increases and almost returns to the original valence state, indicating the oxidation of the sulfur species. The changes of Mo 3d during the charging and discharging process are shown in Figure 7b,e,h. During the discharging process, the valence state of Mo in the AlMo_4_S_8_/CNTs@S was elevated, showing that the interaction of Mo with the formed sulfur species can inhibit the shuttling of polysulfides, thereby improving the cycling stability. Furthermore, the Al 2p peak positions remained stable during charge and discharge (Figure 7c,f,i). Notably, the intensity of the Al 2p XPS peak increased during discharge, indicating that Al participates in the sulfur conversion reaction (Figure S28, Supporting Information).^[^ ^58^ ^]^

**Figure 8:**
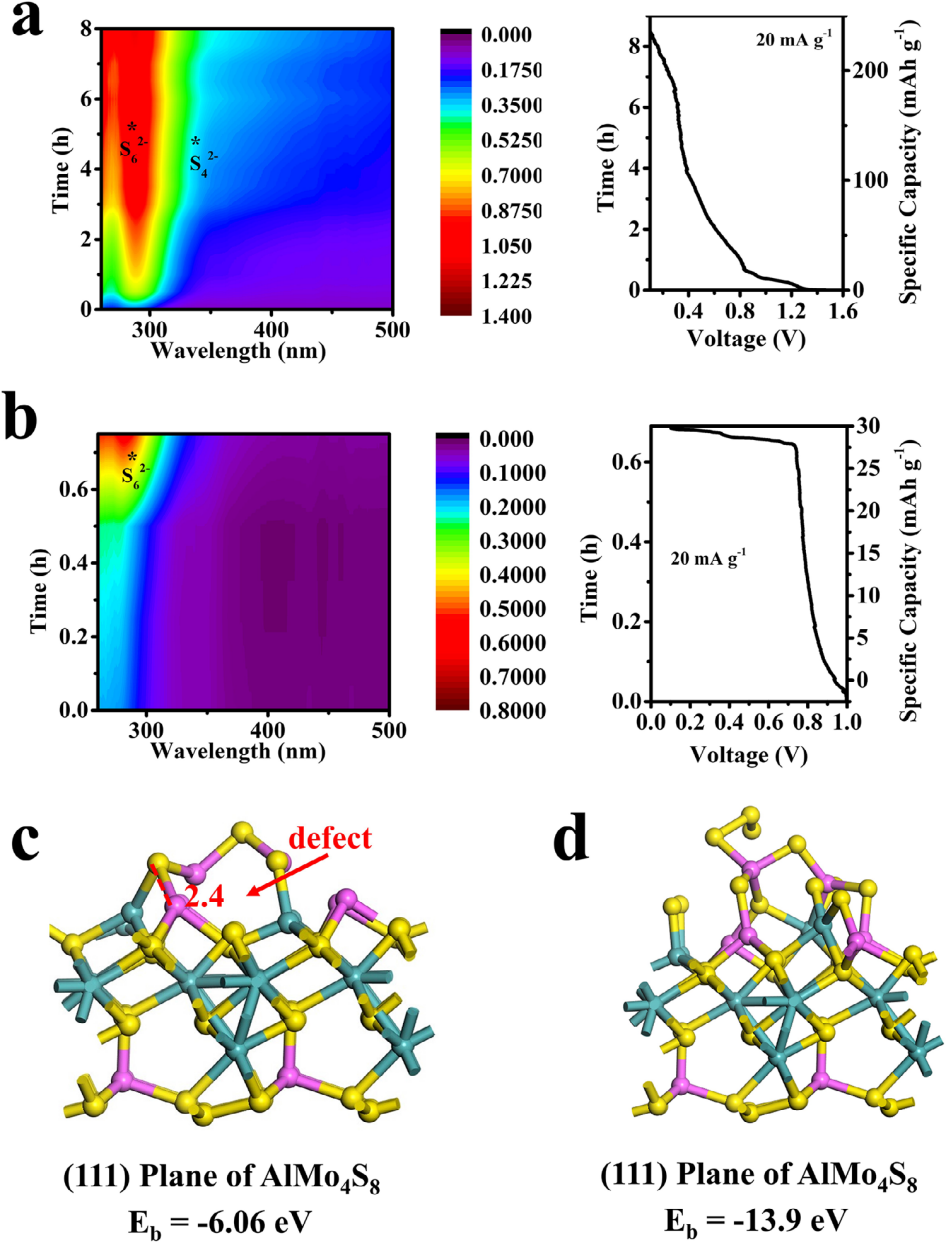
a,b) Contour maps (left) of in situ UV–vis spectra and the corresponding discharge profile (right) with a) AlMo_4_S_8_/CNTs composite S host and b) S; The optimized geometric structures viewed from the side, along with their binding energies of the AlMo_4_S_8_‐Al_2_S_3_ c), AlMo_4_S_8_‐Al_2_S_12_ d) interaction systems. The Al, Mo, and S are highlighted in red, green, and yellow.

**Figure 9:**
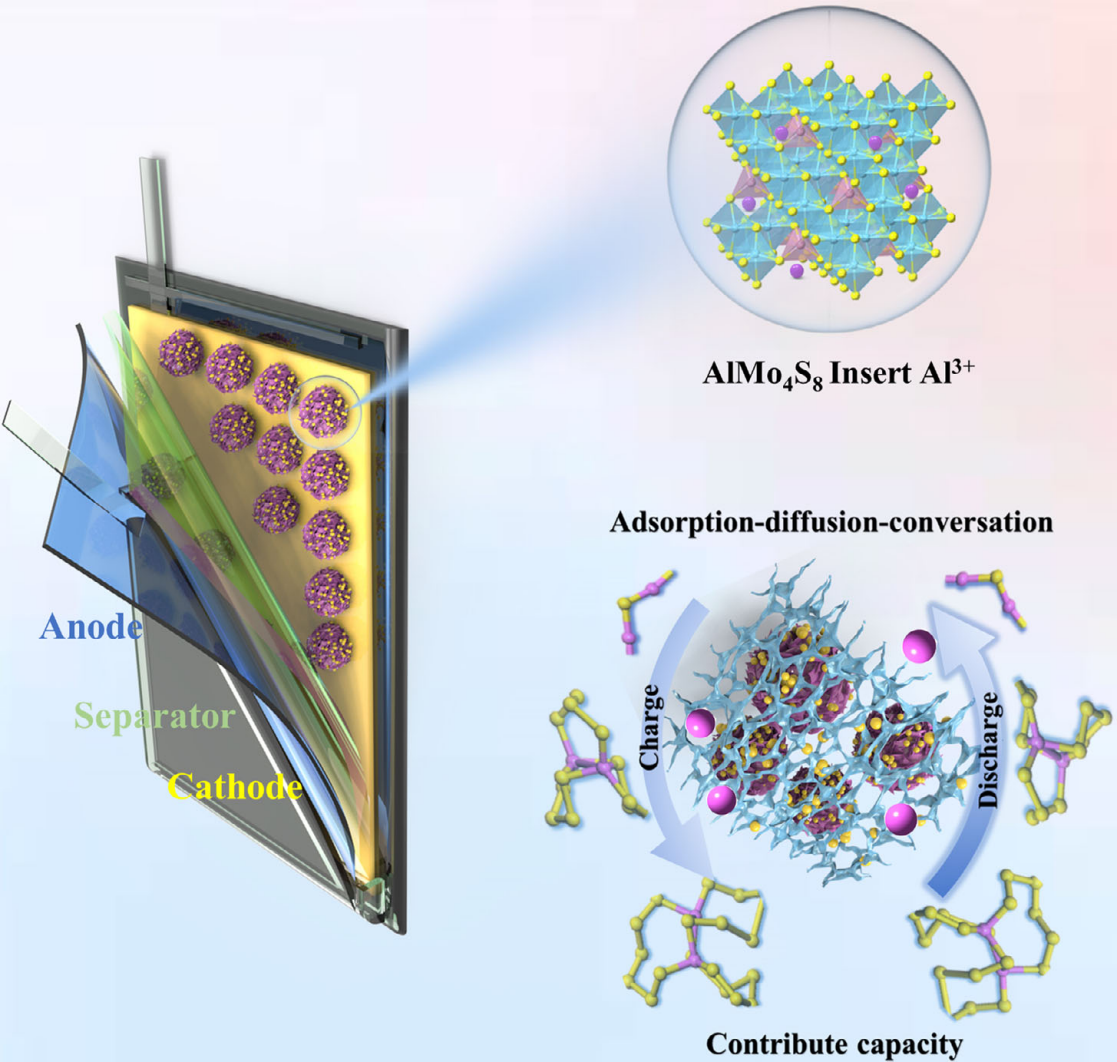
Illustration of inserted Al^3+^ and interaction with AlPSs during charging and discharging.

To gain a comprehensive understanding of the mechanisms underlying the redox kinetics of AlPSs affected by AlMo_4_S_8_/CNTs, we performed in situ UV–vis electrochemical analyses to monitor the evolution of the sulfur intermediates during the discharge/charge process (**Figure** **8**). The Al–S test‐tube cell, featuring a consistent sulfur loading of 3 mg cm^−2^, was immersed in the ionic liquid electrolyte, employing an initial discharge rate of 20 mA g^−1^ for in situ electrochemical studies. The AlMo_4_S_8_/CNTs@S electrode exhibited a significantly higher discharge capacity of 220 mAh g⁻¹ and a greater open‐circuit voltage of 1.5 V, in contrast to the S electrode, which had an open‐circuit voltage of 1.0 V. This indicates that AlMo_4_S_8_/CNTs effectively catalyze the sulfur‐polysulfide interconversion. Additionally, the spectra of the AlMo_4_S_8_/CNTs@S electrode exhibited higher concentrations of S_6_^2−^ and S_4_^2−^ compared to the S electrode, likely due to the redox reactions between AlMo_4_S_8_ and AlPSs.^[^ ^59^, ^60^ ^]^ The non‐occurrence of S conversion during discharge at the S‐controlled electrode limits the availability of AlPSs intermediates in the electrochemical reaction, leading to poor sulfur utilization and slow reaction kinetics. This mainly explains the enhanced electrochemical performance observed for the AlMo_4_S_8_/CNTs@S cathode in Al–S test‐tube cells compared to the S cathode. To further investigate the chemical interactions between AlMo_4_S_8_ and AlPSs (Al_2_S_n_, n = 3, 12), density‐functional theory (DFT) calculations were performed. Figure 8c,d and Figure S24 (Supporting Information) illustrate the optimized geometries of AlMo_4_S_8_ interactions and their binding energies (Eb) with AlPSs. The top view (Figure S29, Supporting Information) and side view (Figure 8c,d) reveal that the (111) face of AlMo_4_S_8_ interacts weakly with Al_2_S_3_, with a chemical binding occurring between the bridging sulfur in Al_2_S_3_ and the Al or Mo cations in AlMo_4_S_8_, resulting in an Eb of −0.6 eV (Figure 8c). The extensive aluminum deficiency within the spinel structure significantly influences the interaction of AlMo_4_S_8_ with both Al_2_S_3_ and Al_2_S_12_. Additionally, the binding energy between the defective AlMo_4_S_8_ with aluminum vacancies and Al_2_S_12_ was −13.9 eV (Figure 8d; Figure S29b, Supporting Information). To further reveal the electronic interactions between Al_2_S_3_, Al_2_S_12_ and AlMo_4_S_8_, the DOS and PDOS of AlMo_4_S_8_∙∙ Al_2_S_12_ and AlMo_4_S_8_∙∙ Al_2_S_3_ were calculated as shown in Figure S30 (Supporting Information). The PDOS position from AlMo_4_S_8_ to AlMo_4_S_8_∙∙∙Al_2_S_3_ and AlMo_4_S_8_∙∙∙Al_2_S_12_ shifts from low energy to high energy. The binding of Al_2_S_3_ to AlMo_4_S_8_ is stronger due to the lower energy and the corresponding higher structural stability.^[^ ^61^, ^62^ ^]^ In contrast, AlMo_4_S_8_∙∙Al_2_S_12_ has poor structural stability and the polysulfide chain is prone to chain breakage. The presence of Al defects leads to chain breakage of the Al_2_S_12_ molecule, which provides a stronger affinity for capturing polysulfides. Thus, the DFT results suggest that the AlMo_4_S_8_ exhibits a strong polysulfide adsorption capacity, particularly around vacancies. **Figure** **9** presents the de‐embedded/insertion of Al^3+^ in AlMo_4_S_8_ vacancies during charge and discharge, along with the interconversion of AlPSs.

The SEM images for anode Al before and after cycling are shown in Figure S31 (Supporting Information). The Al surface exhibits uneven Al grains. After the Al plating/stripping process the aluminum grains disappeared with a smooth surface. The evolution of the AlMo_4_S_8_/CNTs@S morphology after cycling at a current density of 300 mA g^−1^ was observed using SEM and TEM (Figures S32 and S33, Supporting Information). The after‐cycling carbon tubes contracted into the AlMo_4_S_8_ lamellar structure (Figure S34a,b, Supporting Information). The AlMo_4_S_8_/CNTs@S morphology after cycling (Figure S34d–g, Supporting Information) displayed the existence of Al, Mo, S, and C elements on the electrode, which is consistent with the morphology of the original sample. Similarly, AlMo_4_S_8_@S morphology remained sheet‐like after cycling, indicating good cycling stability (Figure S35, Supporting Information). Additionally, incorporation of carbon tubes enhances conductivity, provides a large specific surface area for active sites, shortens the transport distance, buffers the volume change, and contributes to the fast Al^3+^/e^−^ transfer.

## 3 Conclusion

In summary, we propose a hybrid cathode that integrates converted sulfur with embedded AlMo_4_S_8_. The AlMo_4_S_8_ not only enhances its capacity but also exhibits strong chemical interactions with the AlPSs due to the influence of Al ions during charging. DFT calculations, combined with in situ spectroscopic and electrochemical techniques, demonstrate that AlMo_4_S_8_/CNTs effectively confine AlPSs shuttling, catalyze redox kinetics, and achieve a higher specific discharge capacity compared to the sulfur electrode in Al–S cells. The AlMo_4_S_8_/CNTs@S cathode shows a high initial discharge capacity (859.7 mAh g^−1^ at 300 mA g^−1^) and good electrochemical performance (304.3 mAh g^−1^ at 500 mA g^−1^). At extreme temperatures, the AlMo_4_S_8_/CNTs@S cathode exhibited excellent electrochemical performance (911.5 mAh g^−1^ at 50 °C at 300 mA g^−1^).

## Conflict of Interest

The authors declare no conflict of interest.

## Supporting information



Supporting Information

## Data Availability

Data sharing is not applicable to this article as no new data were created or analyzed in this study.
