# Supplementary material for: Defect Spinel Aluminum Molybdenum Sulfide: A Dual‐Function Catalyst for Polysulfide Conversion and Aluminum Intercalation in Aluminum–Sulfur Batteries
Source: Adv Sci (Weinh). 2025 Mar 24;12(19):2417061. doi: 10.1002/advs.202417061 (PMC12097064; doi:10.1002/advs.202417061)
Supplement: Supplementary file 1 — Supporting Information [file ADVS-12-2417061-s001.docx]

Supporting Information

**Defect Spinel Aluminum Molybdenum Sulfide: A Dual-Function Catalyst for Polysulfide Conversion and Aluminum Intercalation in Aluminum-Sulfur Batteries**

Qiuping Zhou,^a^ Yi Liu,^a^ Huayan Zhang,^a^ Chenlong Feng,^a^ Xinyuan Jiang,^a^ Guang Yang,^a^ Yongjun He, ^a^ Ming Chen, ^a^ Guowang Diao,*^a^ Lubin Ni*^a^

**Experimental Section**

**AlMo_4_S_8_ and AlMo_4_S_8_/CNTs material preparation**

A mixture of 150 mg Al_2_S_3_, 152 mg Mo, 30 mg carbon nanotubes and 80 mg sulfur was milled for half an hour by solid-state reaction at 900 °C for 36 h. The obtained powder was then stirred in dimethyl sulfoxide for 6 h. Finally, the powder was centrifuged and dried at 60 °C. Synthesis of AlMo_4_S_8_ above steps without carbon nanotubes.

**Prepared of** **AlMo_4_S_8_/CNTs@S and AlMo_4_S_8_@S electrode materials**

To prepare AlMo_4_S_8_/CNTs@S and AlMo_4_S_8_@S composites, the synthesized AlMo_4_S_8_/CNTs and AlMo_4_S_8_ were mixed with sublimated sulfur in a mass ratio of 3:2 and heated to 155 °C for 20 h.

**Ionic Liquid Electrolyte Preparation**

The ionic liquid electrolyte was acquired by slowly dissolving anhydrous aluminum chloride (AlCl_3_, Sigma-Aldrich, 99.999%) into 1-ethyl-3-methylimidazole ([EMIM]Cl, TCI-Japan, 97%) with a molar ratio 1.3:1 in an argon-atmosphere glove box ([O_2_] < 0.01 ppm, [H_2_O] < 0.01 ppm).

**Materials and characterizations**

The crystal structure of samples was characterized by X-ray powder diffraction (XRD, Bruker D8 super speed, Cu Kα radiation, λ = 1.5406 Å, tube voltage and current: 40 kV, 40 mA). The lattice parameters of samples were determined by high resolution transmission electron microscopy (HRTEM, Tecnai, G2F30 S-TWIN, USA) equipped with an energy dispersive X-ray spectrometer (EDS). The morphology and microstructure were analyzed by scanning electron microscopy (FESEM, Hitachi S-4800 II field emission scanning electron microscopy, 20 kV) and transmission electron microscopy (TEM, Philips TECNAI 12 transmission electron microscopy). Surface chemical composition and element valence state of samples were studied by X-ray photoelectron spectroscopy (XPS, Thermo Science, ESCALAB 250Xi, USA). Carbon content was analyzed by thermogravimetric analysis (Pyris 1 TGA, PerkinElmer) from room temperature to 800 °C at a rate of 10 °C min^-1^ in air flow. Specific surface area and pore size distribution of the materials were determined by N_2_ adsorption analyzer (BET, Micromeritics, TriStar 3000, USA).

**In Situ Visual Vial Cell Assembly**

The AlMo_4_S_8_/CNTs@S and S cathodes were mixed with Super P and PVDF (6:3:1 in weight), Al metal as the anode, and the molybdenum foil as the collector (1 × 2 cm^2^). The sulfur loading was ∼4 mg cm^–2^.

**Electrochemical measurements**

As for the cathodes, the as-prepared samples were ground together with super p and polyvinylidene fluoride (PVDF) with a mass ratio of 6:3:1. With N-methyl-2-pyrrolidone (NMP) as a dispersing agent, the mixture slurry was coated onto a rounded molybdenum current collector (20 mm × 20 mm, 0.05 mm in thickness) and dried at 50 °C under vacuum for 12 h. The loaded amount of active material was about 1.0 mg cm^-2^. ASBs were assembled using a soft pack cell in an argon-filled glove box at room temperature. Aluminum foil (20 mm × 20 mm, 0.05 mm in thickness) served as an anode, Glass fiber paper (GF/D, whatman) were placed between Al anode and cathode. Galvanostatic charge/discharge (GCD) performances was tested on Neware Battery Tester. Cyclic voltammetry (CV) measurements were performed on a CHI 660E electrochemical workstation. Electrochemical impedance spectroscopy (EIS) was conducted on the Autolab-PGSTATA30 workstation at open circuit potential with 10^-2^-10^5^ Hz frequency range and 5 mV alternating current signal amplitude.

**Computational Methods**

We have employed the Vienna Ab initio Simulation Package (VASP) to perform all density functional theory (DFT) calculations within the generalized gradient approximation (GGA) using the Revised Perdew-Burke-Ernzerhof (r-PBE) functional. We have chosen the projected augmented wave (PAW) potentials to describe the ionic cores and take valence electrons into account using a plane wave basis set with a kinetic energy cutoff of 350 eV. The convergence criteria of energy and force were set to 10^−5^ eV and 0.05 eV Å^−1^, respectively. The free surfaces of the AlMo_4_S_8_ (111) slab were separated by a 15 Å vacuum layer. A Monkhorst-Pack k-points of 2×2×1 was applied for all the calculations. The binding energy was calculated as E_b_= E_AlMo4S8/Al2Sx_ − E_Al2Sx_− E_AlMo4S8_, where E_AlMo4S8/Al2Sx_ is the energy of Al_2_S_x_ (x= 3 or 12) and AlMo_4_S_8_ interaction system and E_Al2Sx_ and E_AlMo4S8_ are the energies of the free Al_2_S_x_ and AlMo_4_S_8_, respectively.


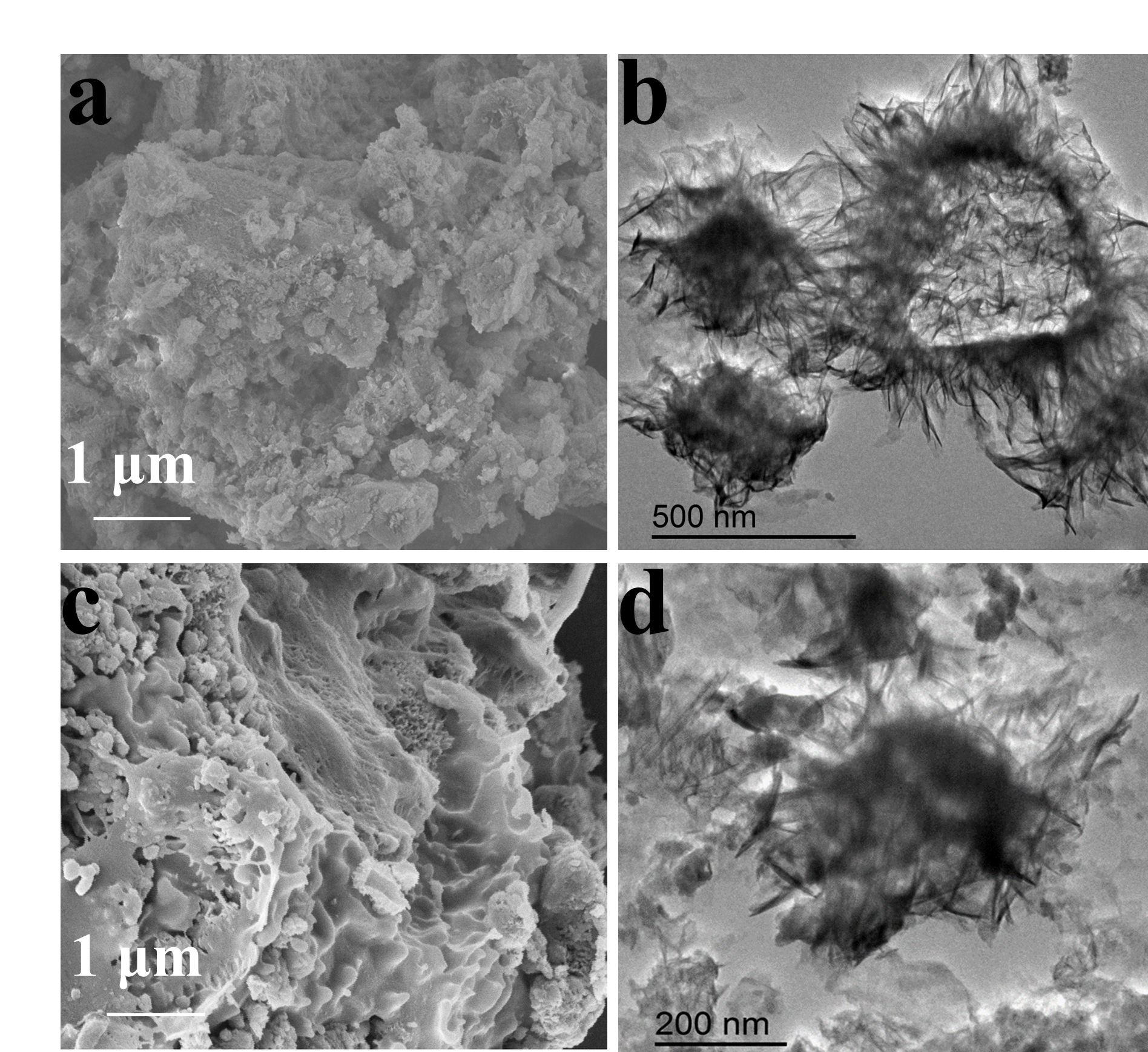


**Figure S1** (a) SEM image, (b) TEM image of AlMo_4_S_8_.


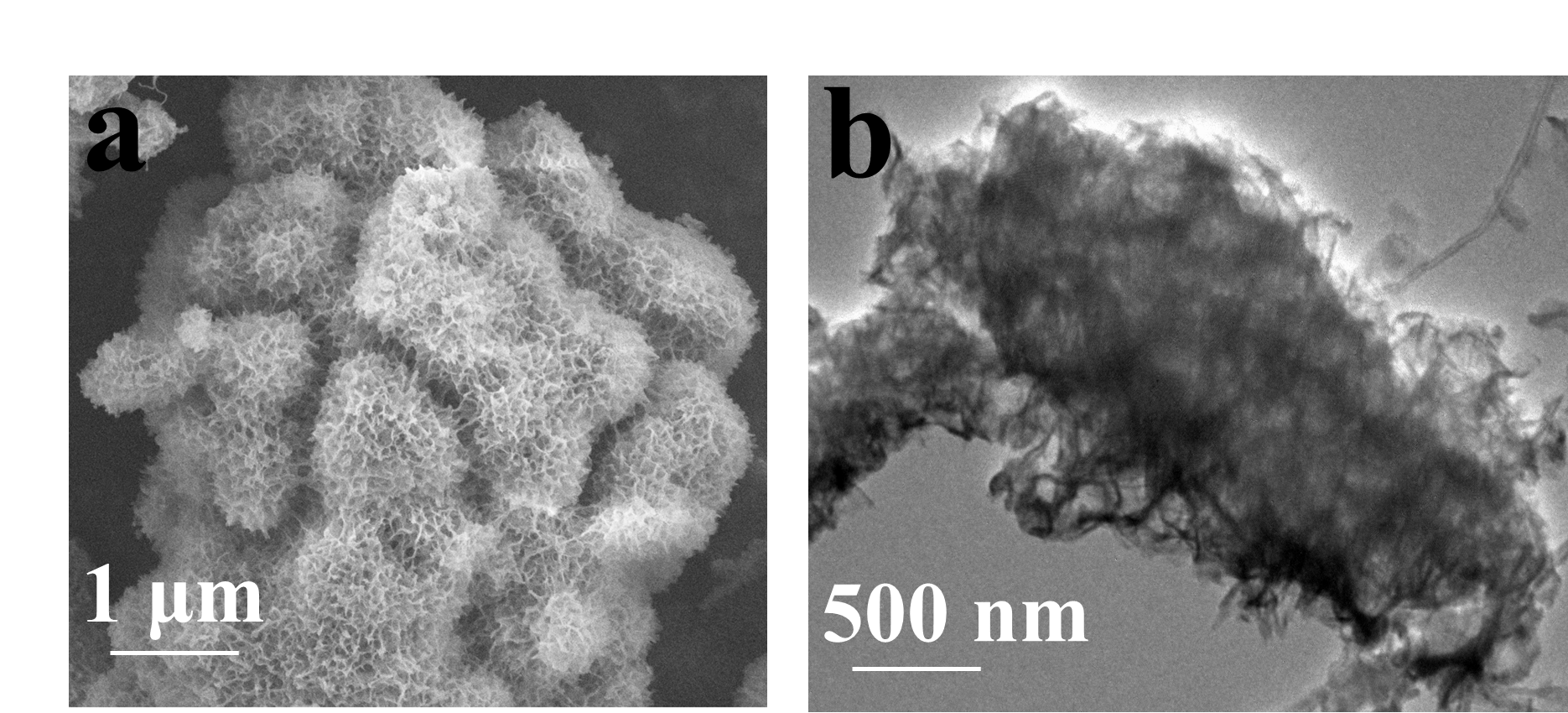


**Figure S2** (a) SEM image, (b) TEM image of AlMo_4_S_8_/CNTs.


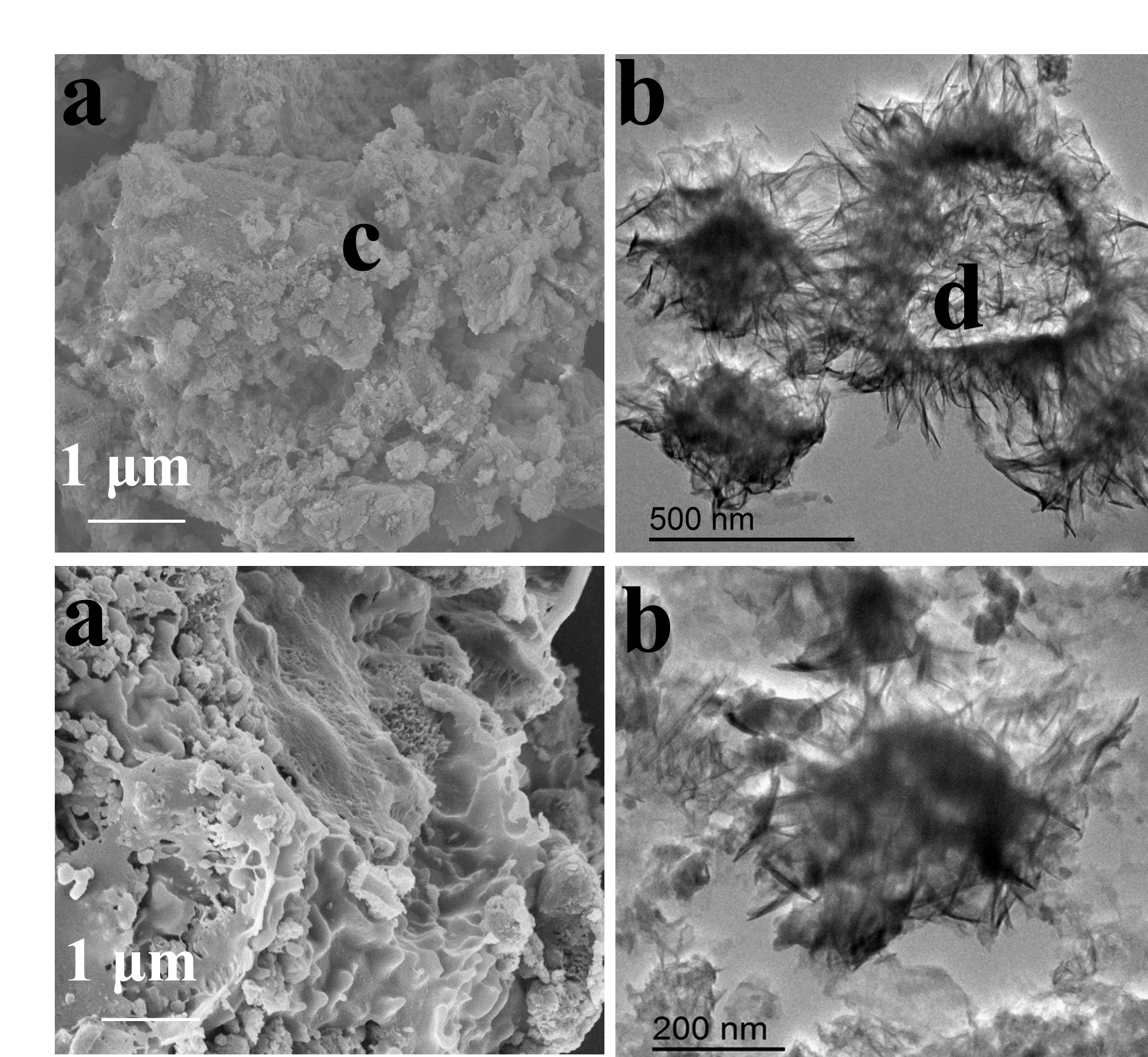


**Figure S3** (a) SEM image, (b) TEM image of AlMo_4_S_8_@S.

**Figure S4** EDS for AlMo_4_S_8_/CNTs@S.


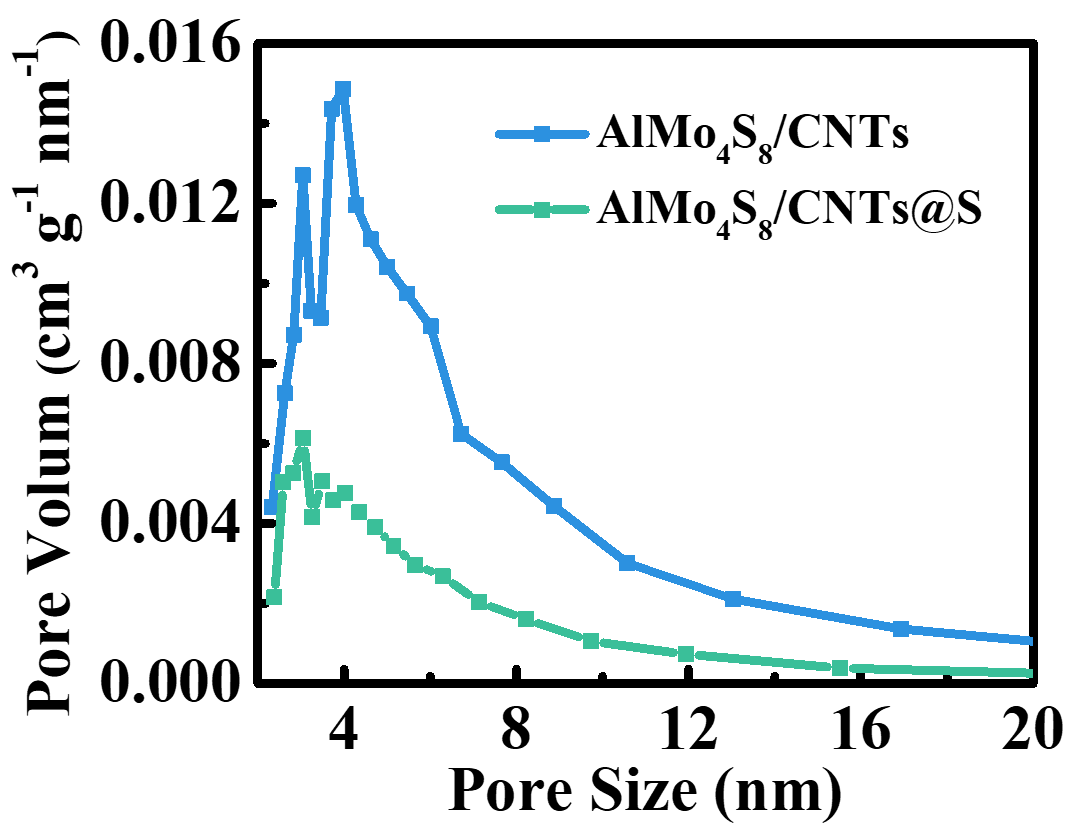


**Figure S5** Pore size distribution curves of AlMo_4_S_8_/CNTs and AlMo_4_S_8_/CNTs@S.


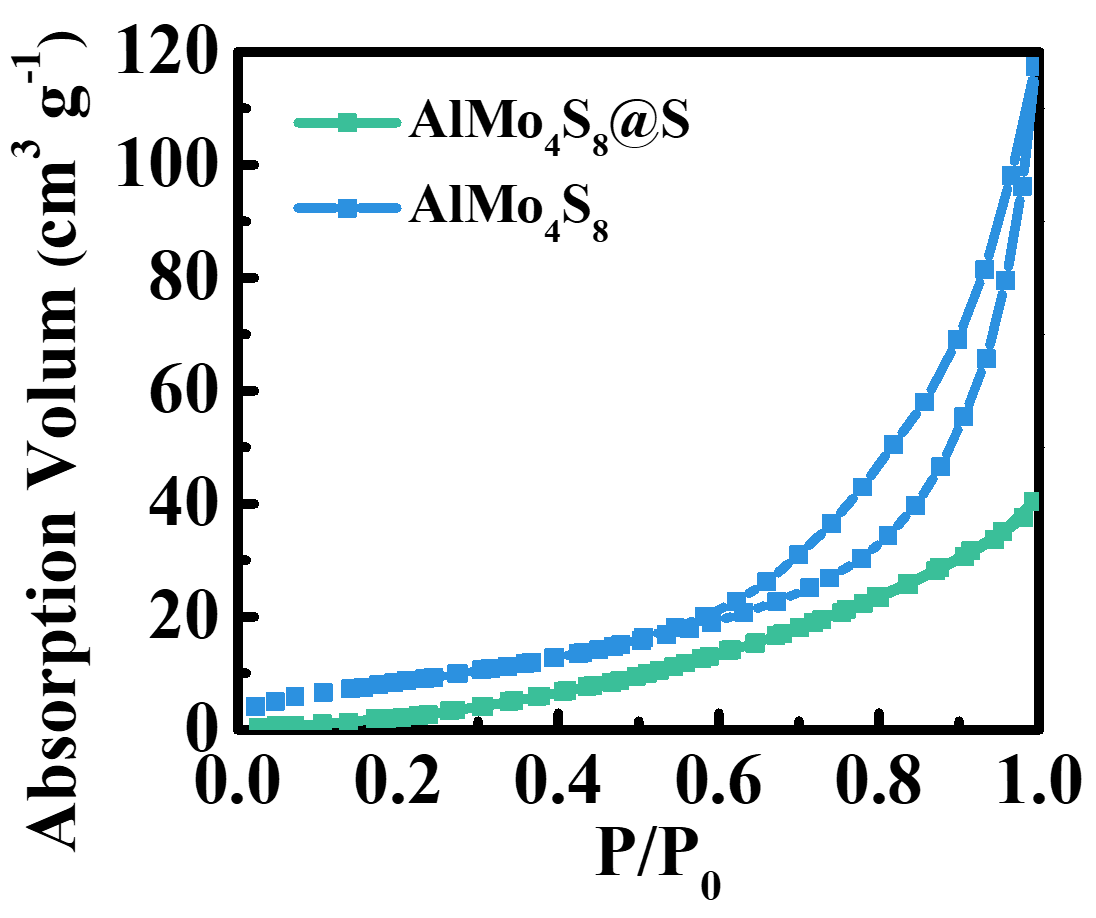


**Figure S6** N_2_ adsorption-desorption isotherms for AlMo_4_S_8_ and AlMo_4_S_8_@S.


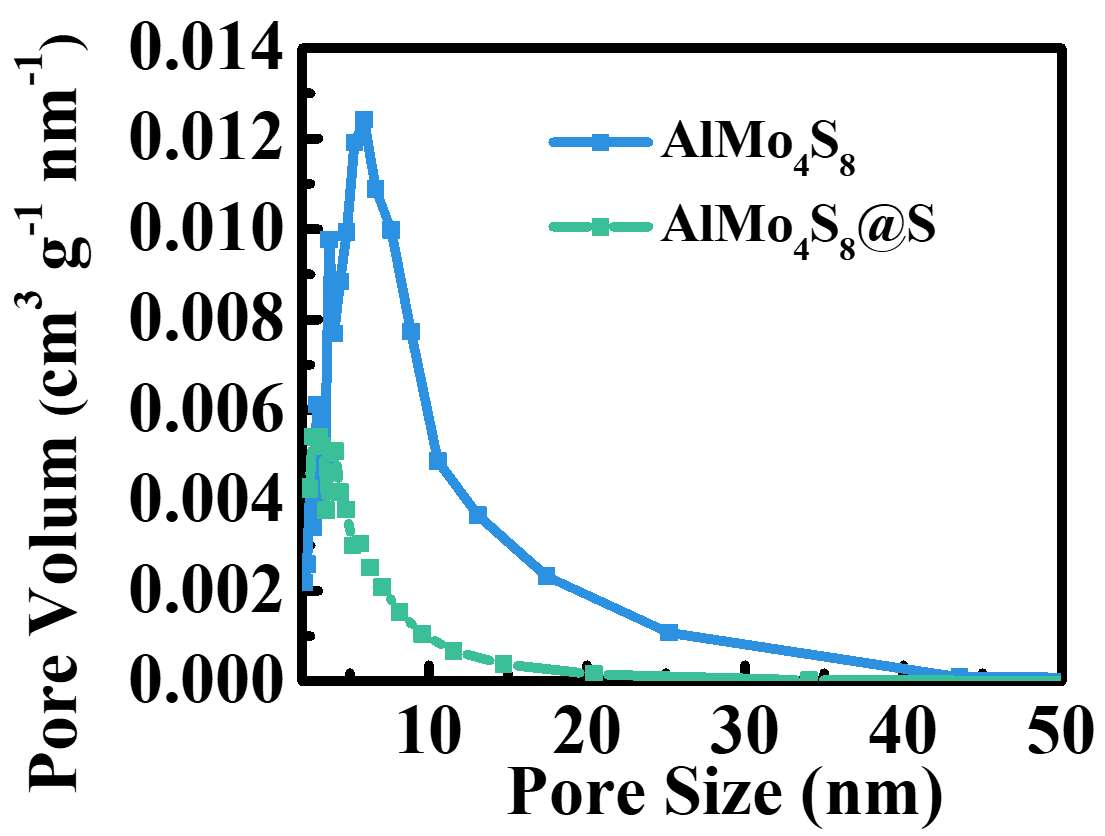


**Figure S7** Pore size distribution curves of AlMo_4_S_8_ and AlMo_4_S_8_@S.

**Figure S8** XPS spectra of Mo 3d from AlMo_4_S_8_.

**Figure S9** XPS spectra of S 2p from AlMo_4_S_8_.

**Figure S10** XPS spectra of Al 2p from AlMo_4_S_8_.


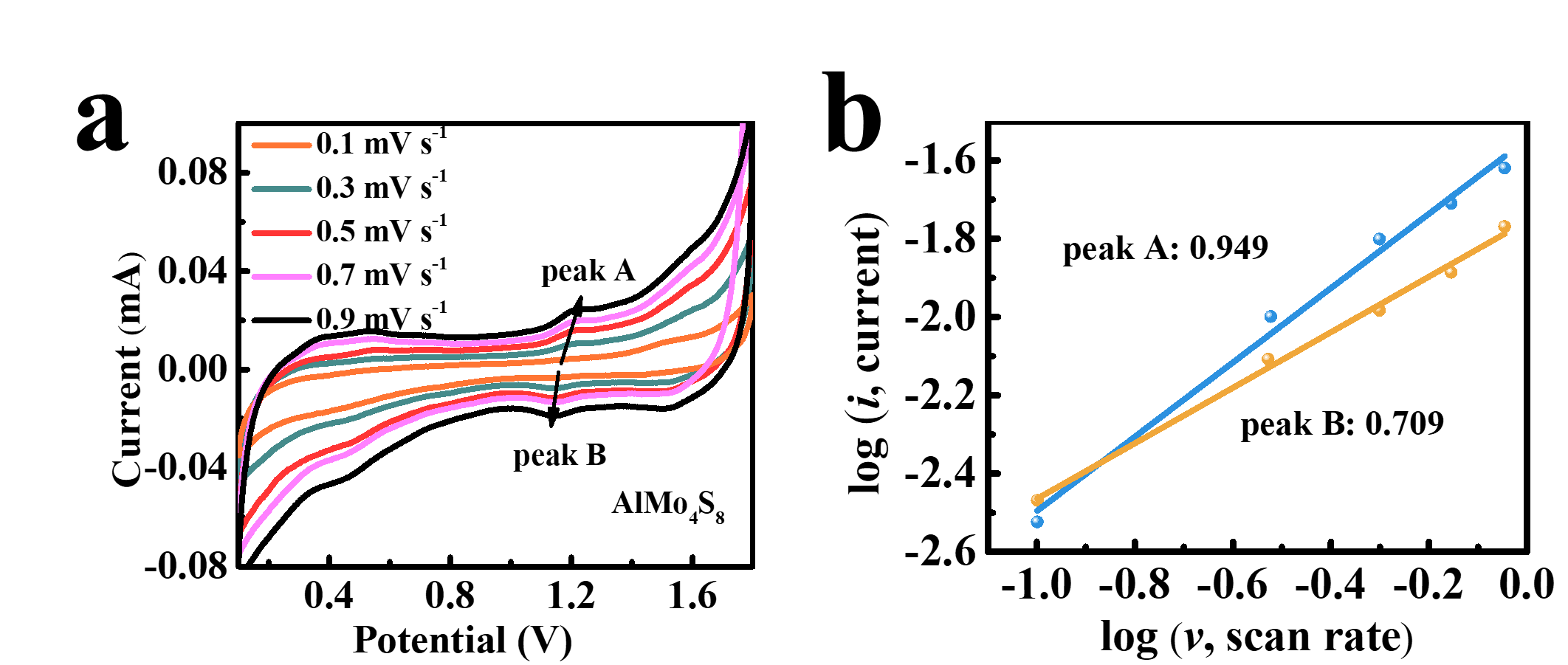


**Figure S11** CV curves at different scan rates (0.1, 0.3, 0.5, 0.7 and 0.9 mV s^-1^) a); B-value determination b).


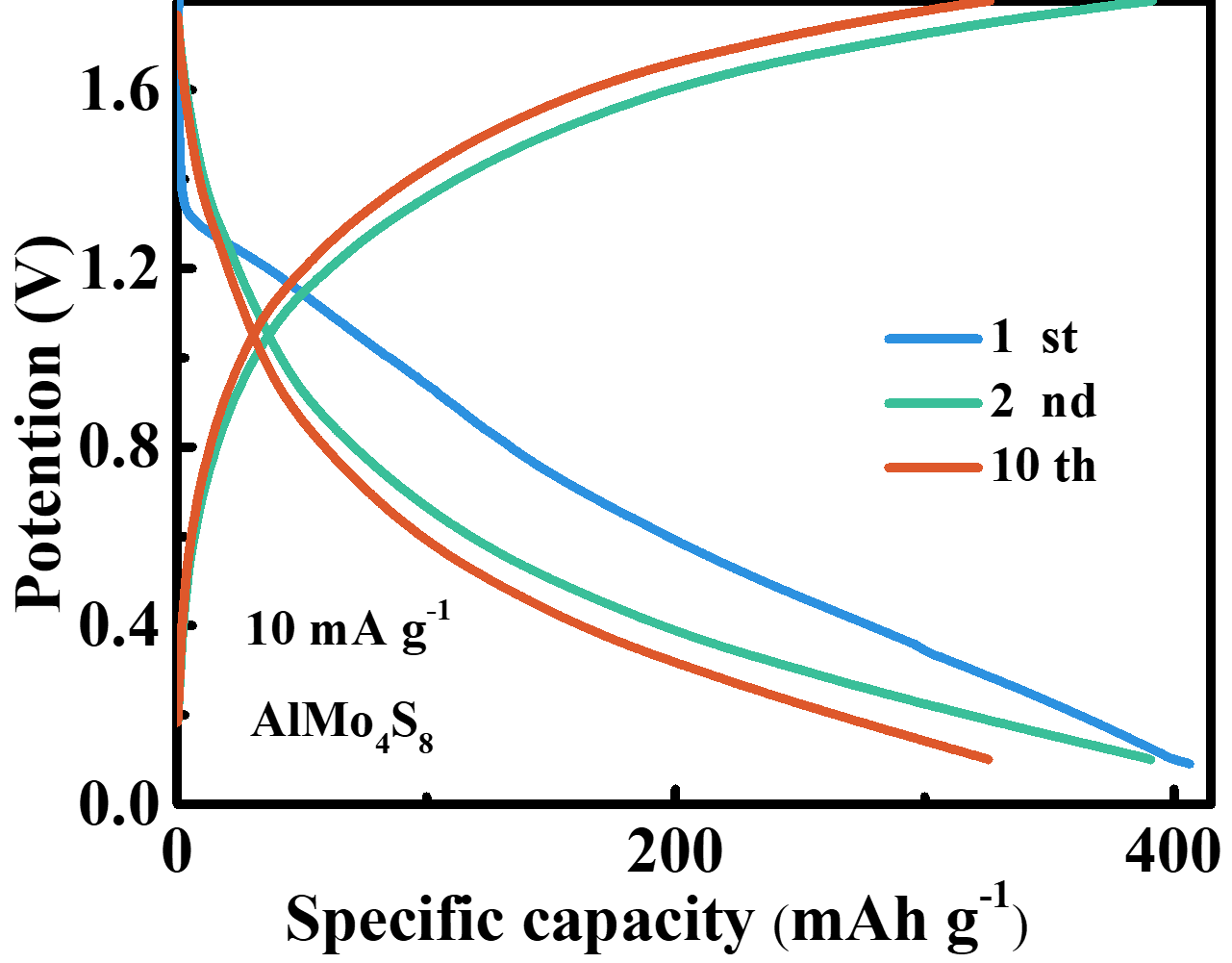


**Figure S12** The charge-discharge curve of Al-ion battery with AlMo_4_S_8_ at 10 mA g^-1^.

**Figure S13** Cycling performance of AlMo_4_S_8_ at 500 mA g^-1^.


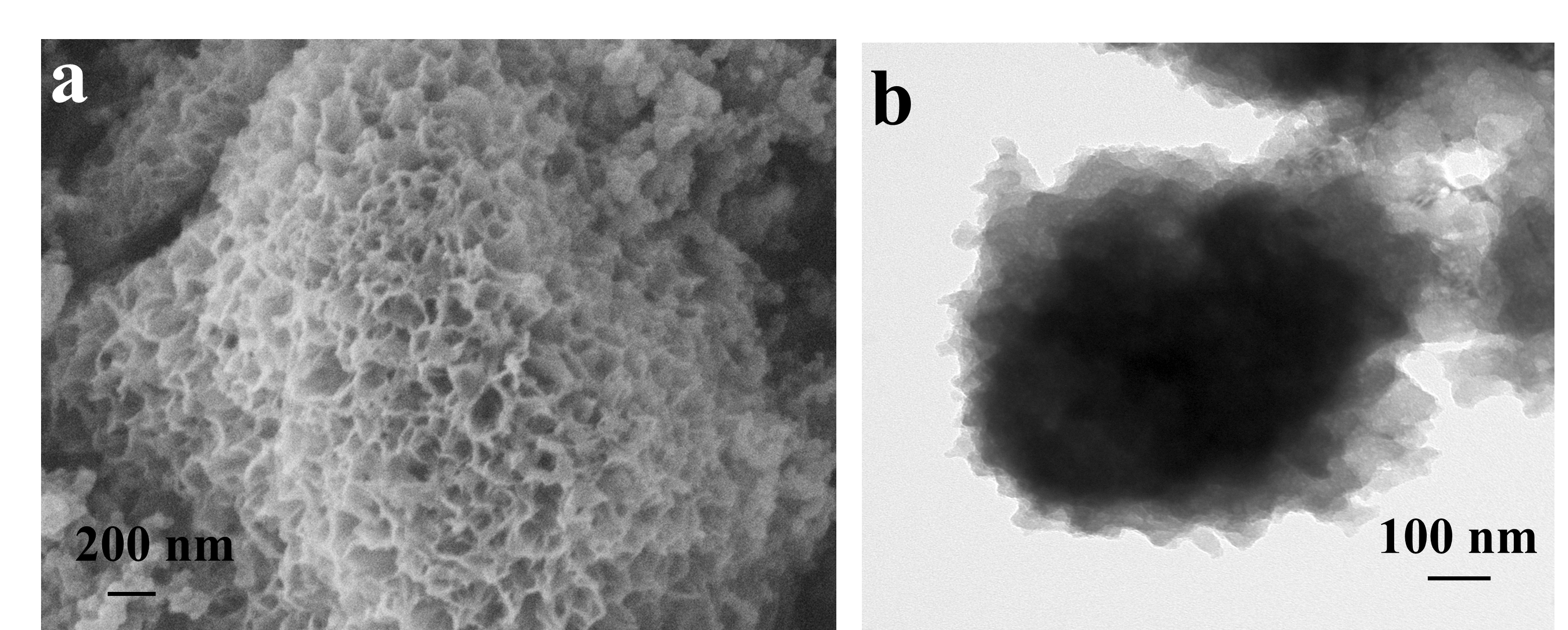


**Figure S14** SEM and TEM images of AlMo_4_S_8_ after cycling.

**Figure S15** Nyquist plots of AlMo_4_S_8_//Al.

**Figure S16** XPS spectra of Al 2p from cycling AlMo_4_S_8_.


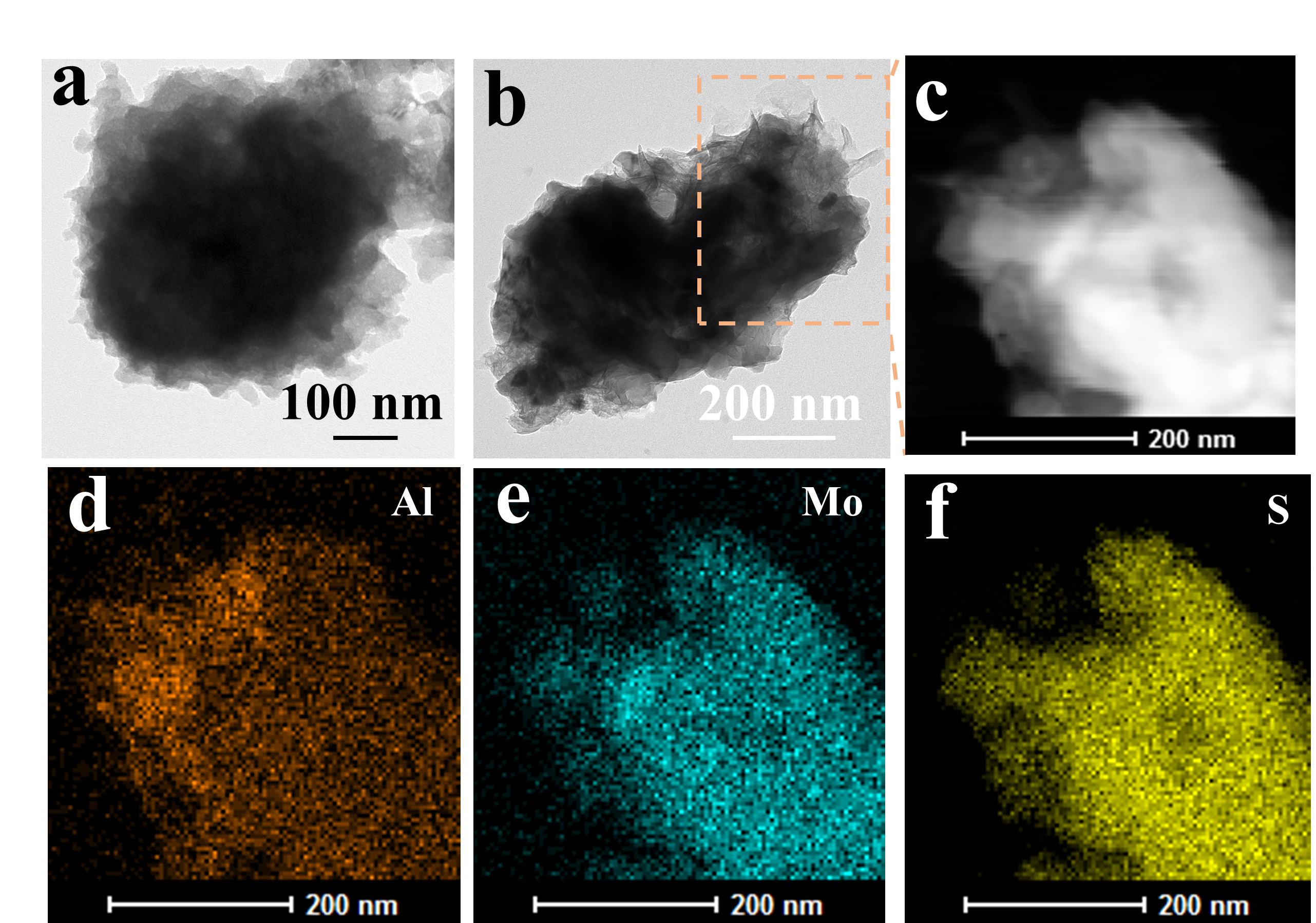


**Figure S17** (a, b) TEM images of AlMo_4_S_8_ after cycling; (c) STEM images of AlMo_4_S_8_ after cycling; (d-f) Elemental mapping of AlMo_4_S_8_ composite on Al, Mo, and S.


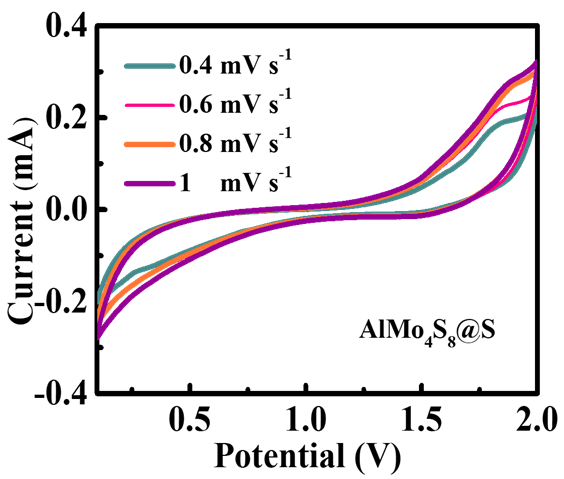


**Figure S18** CV curves of AlMo_4_S_8_@S at different scan rate.

**Figure S19** Cycling performance of AlMo_4_S_8_@CNTs@S at 100 mA g^-1^.


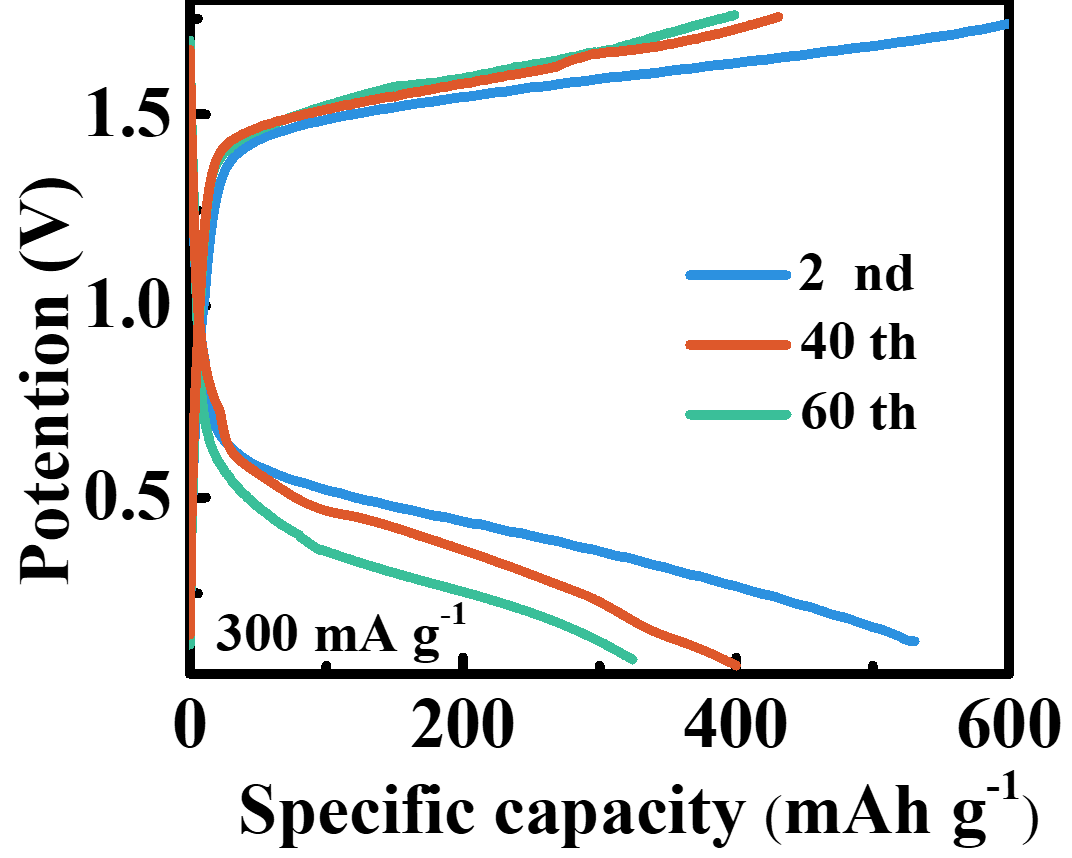


**Figure S20** The discharge/charge curves for AlMo_4_S_8_@CNTs@S at 300 mA g^-1^.

**Figure S21** The discharge/charge curves for AlMo_4_S_8_@CNTs@S at 500 mA g^-1^.

**Figure S22** The charge and discharge curves at different current densities for AlMo_4_S_8_@CNTs@S.

**Figure S23** Cycling performance of CNTs@S.

**Figure S24** Cycling performance of ASBs at large load S.

**Table S1** Comparison of the cyclic stability of our work with previously reported sulfur-based cathodes in ASBs.

| Materials | Last cycle discharge capacity (mAh g^-1^) | | Cycle number | Current density (mA g^-1^) | Ref. |
| --- | --- | --- | --- | --- | --- |
| ACC/S | | 1200 | 6 | 50 | 1 |
| SPAN | | 201 | 22 | 25 | 2 |
| HC/S | | 378 | 28 | 100 | 3 |
| CoNG/S | | 500 | 10 | 200 | 4 |
| TiN@NG/S | | 500 | 200 | 100 | 5 |
| AlMo_4_S_8_/CNTs@S | | 160 | 100 | 500 | This work |

**Figure S25** Nyquist plots of AlMo_4_S_8_@S and AlMo_4_S_8_/CNTs@S.

**
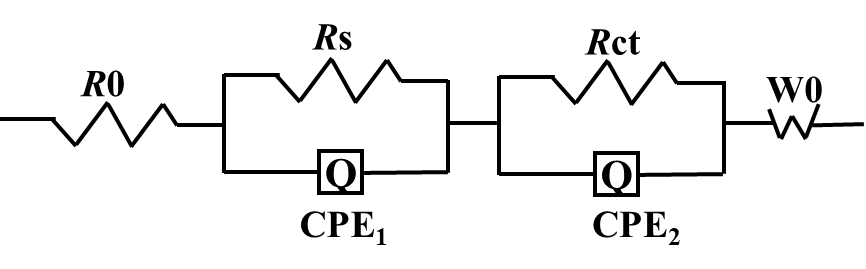
**

**Figure S26** Equivalent circuit model for EIS fitting.

**Table S2** Fitted equivalent circuit elements of AlMo_4_S_8_/CNTs@S and AlMo_4_S_8_@S electrodes

| Sample | *R*_0_ (Ω) | *R*_surf_ (Ω) | *R*_ct_ (Ω) |
| --- | --- | --- | --- |
| AlMo_4_S_8_/CNTs@S | 3.5 | 483 | 232 |
| AlMo_4_S_8_@S | 6.6 | 1599 | 692 |

**Figure S27** XPS spectra of S 2p from cycling electrode S.

**Figure S28** XPS spectra of Al 2p from cycling AlMo_4_S_8_/CNTs@S.


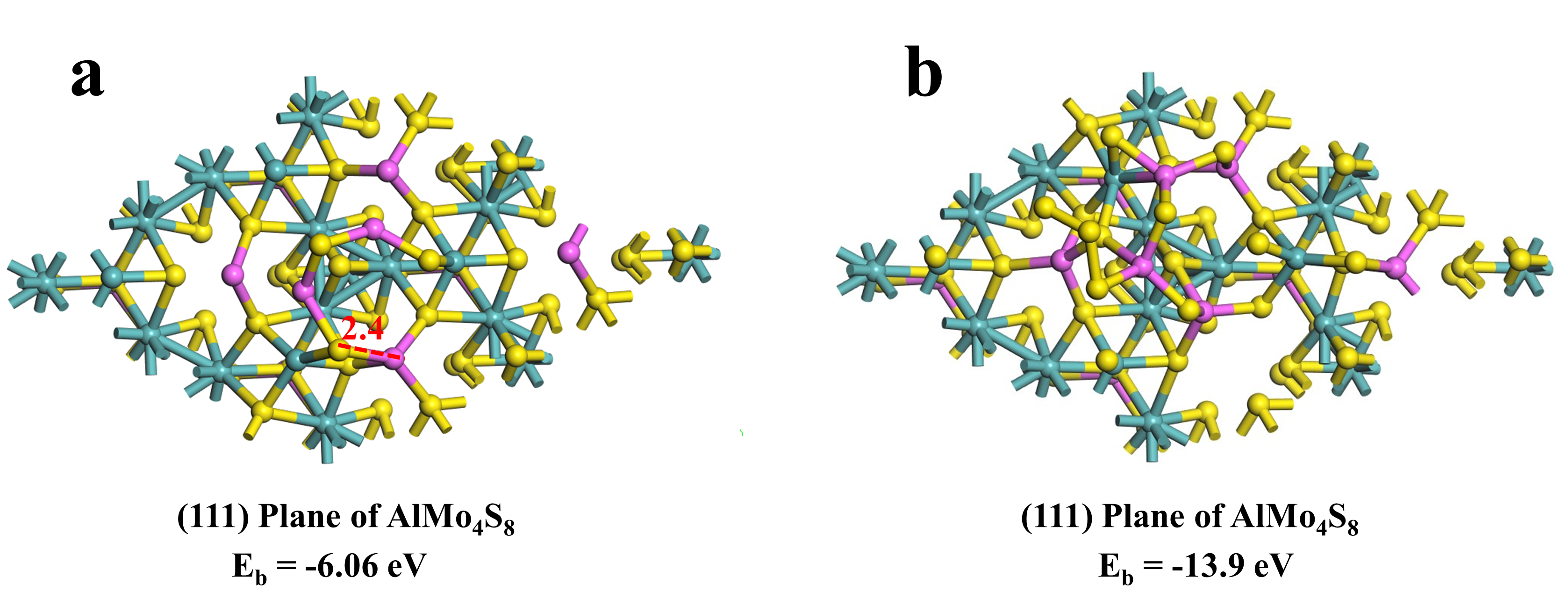


**Figure S29** The top view optimized geometric structures with binding energies of the AlMo_4_S_8_-Al_2_S_3_ a), AlMo_4_S_8_-Al_2_S_12_ b) interaction systems. The change of Al, Mo, and S is marked in red, green, and yellow near the corresponding atoms.


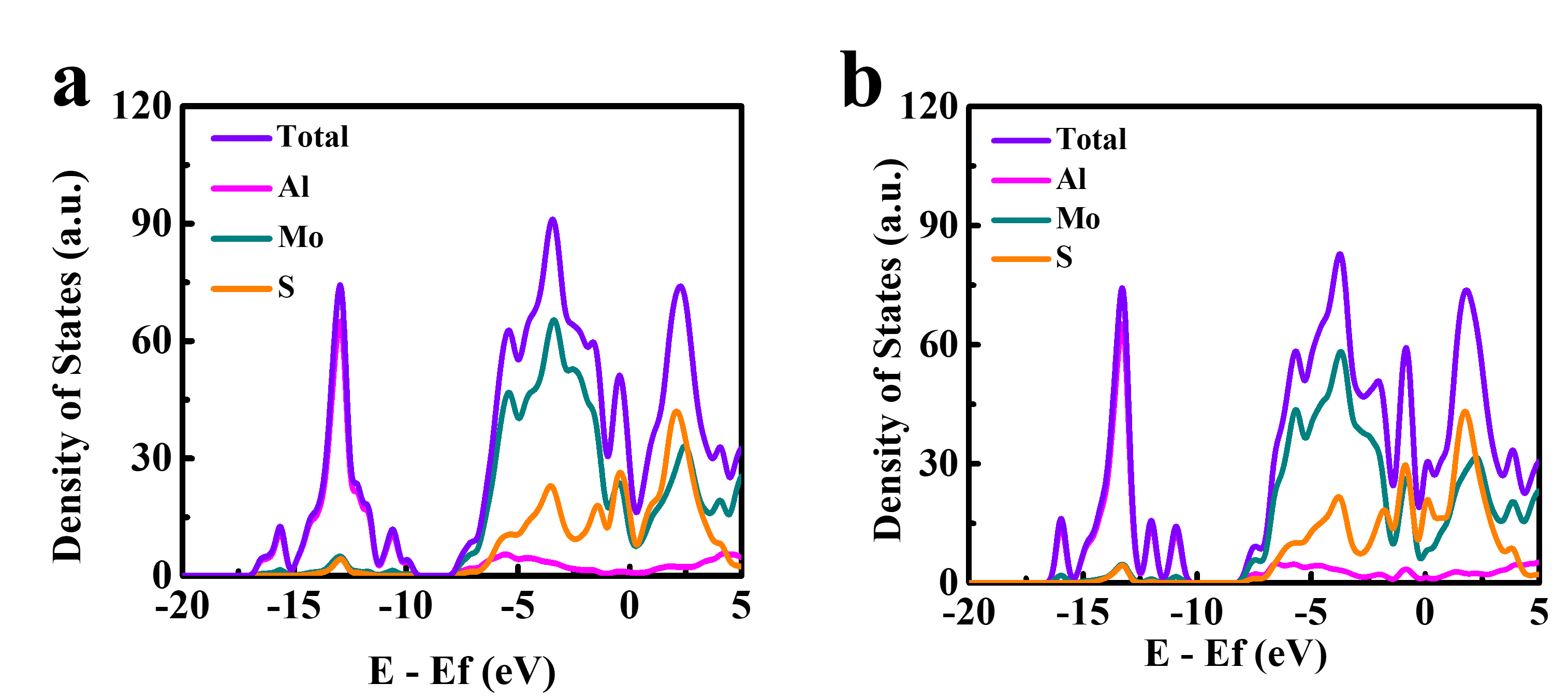


**Figure S****30** The total DOS and PDOS for a) AlMo_4_S_8_∙∙Al_2_S_12_ b) AlMo_4_S_8_∙∙Al_2_S_3_ adsorption configurations.


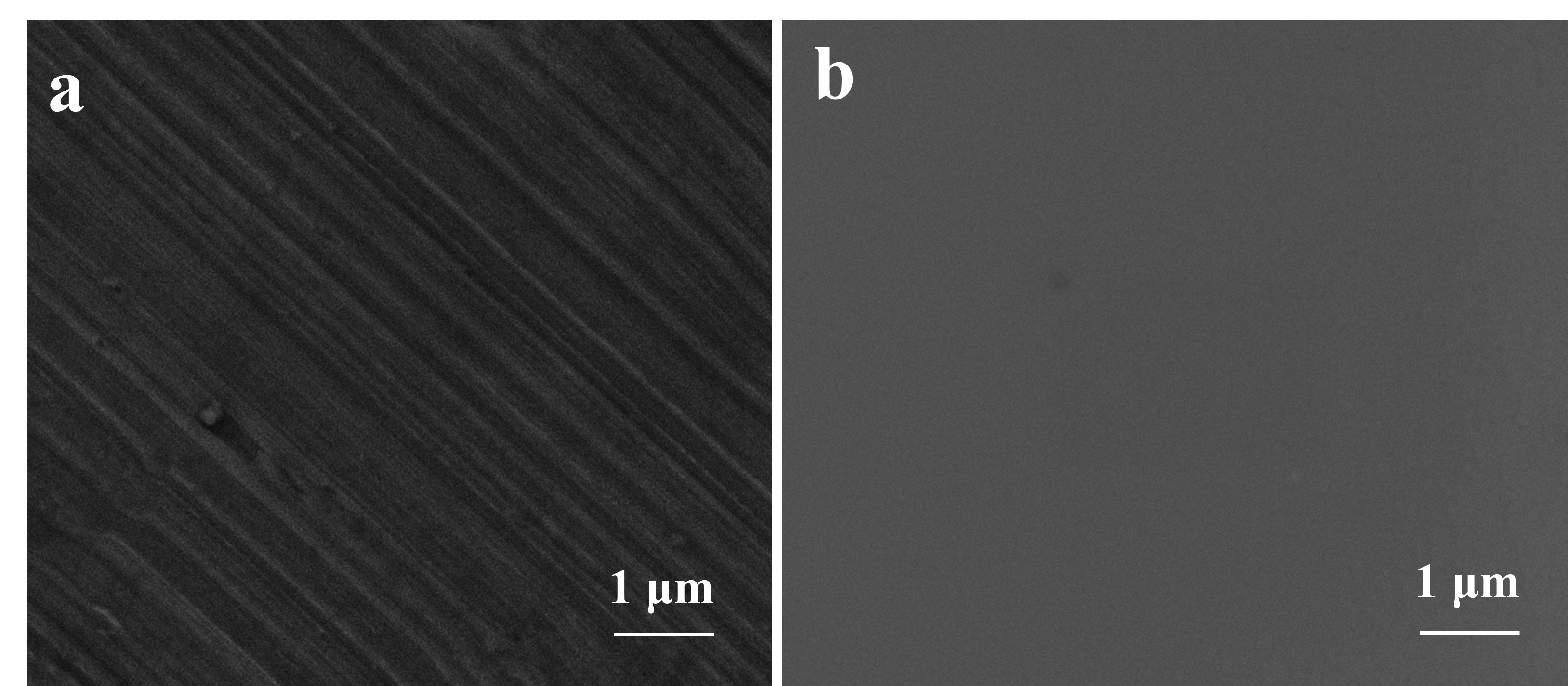


**Figure 31** a, b) SEM images for anode Al before and after cycle.


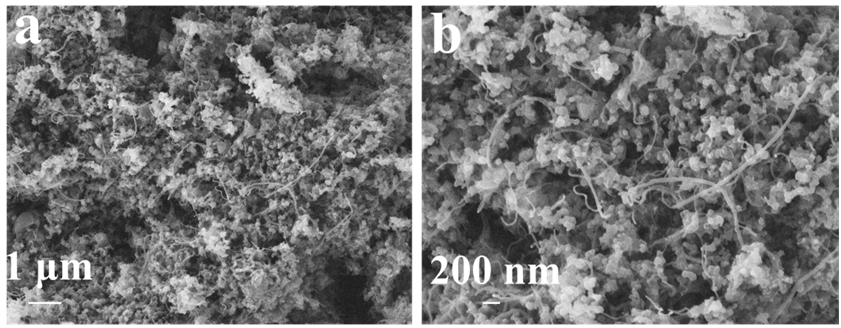


**Figure S32** SEM images of AlMo_4_S_8_/CNTs@S after cycling.


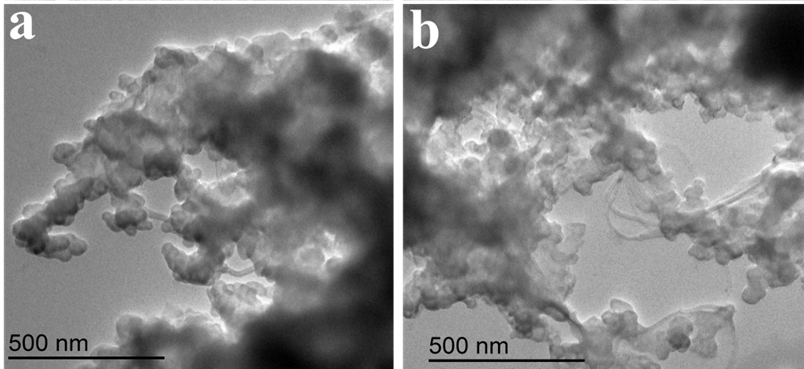


**Figure S33** TEM images of AlMo_4_S_8_/CNTs@S after cycling.


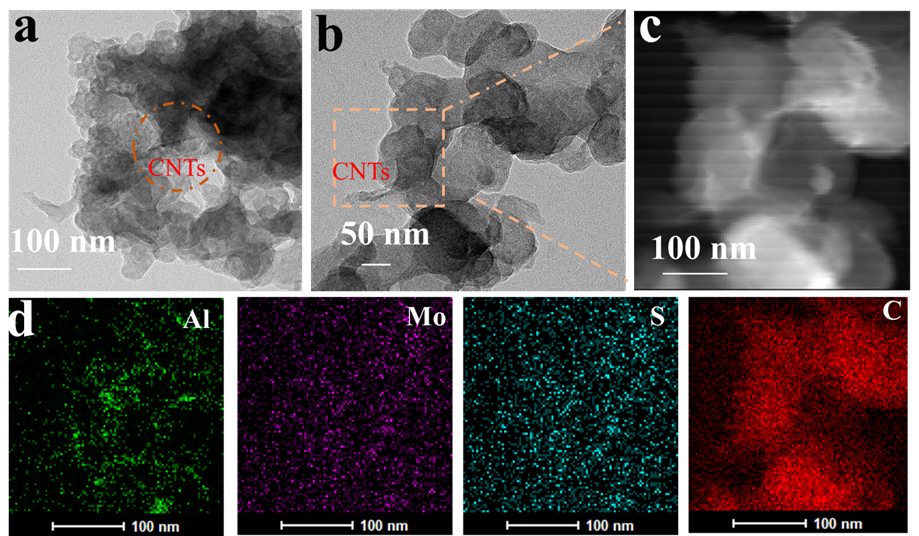


**Figure 34** a-c) TEM and STEM images of cathode AlMo_4_S_8_/CNTs@S after cycling. d-g) Elemental mapping of AlMo_4_S_8_/CNTs@S composite on Al, Mo, S, and C.


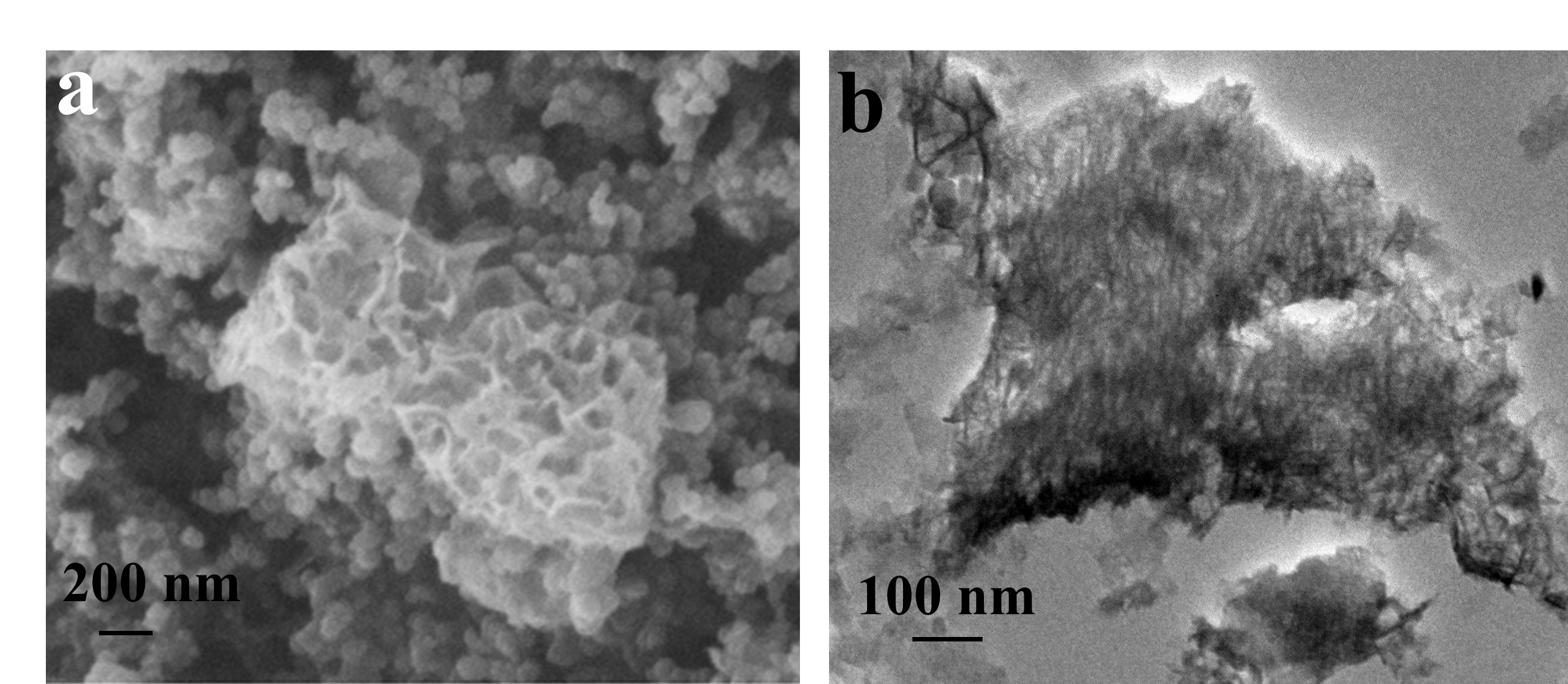


**Figure 35** a, b) SEM and TEM images of cathode AlMo_4_S_8_@S after cycling.

**Reference**

[1] T. Gao, X. Li, X. Wang, J. Hu, F. Han, X. Fan, L. Suo, A.J. Pearse, S.B. Lee, G.W. Rubloff, K.J. Gaskell, M. Noked, C. Wang, Angew. Chemie. 128 (2016) 10052–10055.

[2] W. Wang, Z. Cao, G.A. Elia, Y. Wu, W. Wahyudi, E. Abou-Hamad, A.H. Emwas, L. Cavallo, L.J. Li, J. Ming, ACS Energy Lett. 3 (2018) 2899–2907.

[3] Y. Zhang, L. Ma, R. Tang, X. Zheng, X. Wang, Y. Dong, G. Kong, F. Zhao, L. Wei, Int. J. Hydrogen Energy. 46 (2021) 4936–4946.

[4] Z. Hu, S. Xie, Y. Guo, Y. Ye, J. Zhang, S. Jin, H. Ji, J. Energy Chem. 67 (2022) 354–360.

[5] Y. Ai, S.-C. Wu, F. Zhang, X. Zhang, R. Li, Y. Lan, L. Cai, W. Wang, Energy Storage Mater. 48 (2022) 297–305.
